# A Multidimensional and Integrated Rehabilitation Approach (A.M.I.R.A.) for Infants at Risk of Cerebral Palsy and Other Neurodevelopmental Disabilities

**DOI:** 10.3390/children12081003

**Published:** 2025-07-30

**Authors:** Angela Maria Setaro, Erika Loi, Serena Micheletti, Anna Alessandrini, Nicole D’Adda, Andrea Rossi, Jessica Galli, AMIRA Group, Elisa Fazzi

**Affiliations:** 1 Unit of Child Neurology and Psychiatry, ASST Spedali Civili of Brescia, 25123 Brescia, Italy; angelamaria.setaro@gmail.com (A.M.S.); serena.micheletti@unibs.it (S.M.); anna.alessandrini@unibs.it (A.A.); nicole.dadda@asst-spedalicivili.it (N.D.); andrea.rossi@asst-spedalicivili.it (A.R.); jessica.galli@unibs.it (J.G.); elisa.fazzi@unibs.it (E.F.); 2 Department of Clinical and Experimental Sciences, University of Brescia, 25123 Brescia, Italy

**Keywords:** cerebral palsy, early intervention, enriched environment, adaptive functions, multidimensional approach

## Abstract

**Background/Objectives:** Early experiences can significantly influence brain development, particularly when they occur during specific time windows known as sensitive or critical periods. Therefore, the early promotion of neurodevelopmental functions is crucial in children at risk for neurodevelopmental disabilities, such as those with cerebral palsy. This article introduces AMIRA (A Multidimensional and Integrated Rehabilitation Approach), a rehabilitative framework designed for infants at risk of neurodevelopmental disabilities. **Methods:** AMIRA is intended to guide clinical–rehabilitation reasoning rather than prescribe a rigid sequence of predetermined activities for the child. The theoretical foundation and structure of AMIRA are presented by formalizing its criteria, objectives, tools, and intervention procedures. The framework comprises four distinct sections, each supported by adaptive strategies to facilitate access to materials and to promote play-based interactions among the child, their environment, and communication partners. Particular attention is given to optimizing both micro- and macro-environments for children with, or at risk of, co-occurring visual impairment. Each rehabilitative section includes three progressive phases: an initial observation phase, a facilitation phase to support the child’s engagement, and an active experimentation phase that gradually introduces more challenging tasks. **Results:** The intervention pathways in AMIRA are organized according to six core developmental domains: behavioral–emotional self-regulation, visual function, postural–motor skills, praxis, interaction and communication, and cognitive function. These are outlined in structured charts that serve as flexible guidelines rather than prescriptive protocols. Each chart presents activities of increasing complexity aligned with typical developmental milestones up to 24 months of age. For each specific ability, the corresponding habilitation goals, contextual recommendations (including environmental setup, objects, and tools), and suggested activities are provided. **Conclusions:** This study presents a detailed intervention approach, offering both a practical framework and a structured set of activities for use in rehabilitative settings. Further studies will explore the efficacy of the proposed standardized approach.

## 1. Introduction

Infants at risk of brain damage exhibit a broad spectrum of neurodevelopmental disabilities, often co-occurring, such as cerebral palsy (CP), developmental coordination disorders, intellectual disabilities, psychomotor delay, and sensory impairments [1,2,3], such as Cerebral Visual Impairment (CVI) [4]. The early identification of atypical developmental signs in at-risk infants is a critical starting point for timely care and intervention, with the goal of enhancing brain plasticity, minimizing developmental challenges related to brain dysfunction, and promoting the best developmental potential by capitalizing on sensitive periods of neuroplasticity during early development [5,6,7]. It is well-known that early experiences can profoundly shape brain development [8,9], particularly when these are confined to specific time windows known as sensitive or critical periods [10], which vary for different developing functions, each following distinct yet closely intertwined trajectories of maturation and development. For these reasons, early intervention delivered during the first year of life holds greater potential impact than the same intervention provided later, and should be oriented toward supporting the child’s global neurodevelopment. While focusing on functions particularly at risk based on the child’s clinical profile, early intervention must also address the promotion of all the functions (motor, sensory, cognitive, communicative, social, and regulatory) that are the goals of neurodevelopment [11]. Enhancing one function also means working on those that will develop over a longer or subsequent period of time.

In recent decades, there has been a significant evolution in both the theoretical framework and practical approaches to early intervention, in most cases, focused on the promotion of a specific function [12], in different contexts and addressing different neurodevelopmental disabilities, namely, CP [13], language and communication disorders [14], Autism Spectrum Disorder [15], and visual impairment [16].

Neuroscientific evidence shows that affective, cognitive, and motor processes are deeply interconnected [17], and that early enriched environments, defined as “a combination of complex inanimate objects and social solicitation [18], fosters sensory, cognitive, and motor development [12,19,20], resulting in lasting changes in the central nervous system at the functional, structural, and molecular levels [21]. This underscores the central role of the social context and the caregiver (therapist and/or parent) in creating emotionally supportive relationships that enhance the child’s motivation, engagement, and learning potential, as conceptualized by Vygotsky’s zone of proximal development [22]. By selecting or adapting environmental features to ensure accessibility and usability [23,24], caregivers facilitate active exploration and satisfaction in cognitive and sensorimotor activities. Given that motor behavior originates from intentions shaped by emotions, needs, and social cues [25,26,27], early rehabilitation should be based on goal-directed activities that are meaningful to the child and can be practiced in therapy and generalized to daily routines through parental empowerment [6,28,29,30]. Ultimately, intervention goals should align with the child’s intrinsic motivation to act, explore, and interact, supporting global development through play and meaningful engagement with both people and the environment (e.g., home and nursery).

In this context, we propose the AMIRA approach (A Multidimensional and Integrated Rehabilitation Approach) [31,32,33,34], inspired also by the principles of the Italian Society of Child and Adolescent Neuropsychiatry—SINPIA—and the Italian Society of Physical Medicine and Rehabilitation—SIMFER [35,36]. AMIRA represents a rehabilitation framework specifically designed for children at risk of neurodevelopmental disabilities, with a particular focus on CP, as recently underlined by Dan and colleagues (2025) [37]. Alongside motor impairments, children with CP present with challenges in other functional areas, such as cognition, communication, sensory processing, emotional regulation, and behavior. Early interventions addressed to infants at risk of or with CP should, thus, not only be timely and intensive, but also multidimensional and tailored to the infant’s evolving profile. This approach should also include modifications of the micro- and macro-environment based on the presence and severity level of possible sensory and motor limitations. The AMIRA approach has the goal of enhancing all the neurodevelopmental functions in order to enable individuals to better adapt to their environment, in accordance with individual, social, and cultural context expectations [38] and according to the Ecological Theory [39,40], which posits that movement arises from the interaction between an individual’s physical characteristics and the environment, particularly the pragmatic opportunities it offers—referred to as affordances [39,41,42]. The AMIRA protocol also provides specifications for adapting activities to the various levels of motor and visual limitations. AMIRA is a family-centered approach since the family is an essential and irreplaceable component of the therapeutic process. This includes addressing caregivers’ concerns, challenges, and contributions to the rehabilitation process. By sharing interpretative frameworks of the child’s behavior and transferring knowledge and strategies tailored to their clinical condition, AMIRA aims to foster a sense of competence and effectiveness within the family in caring for the child. Therapeutic interventions that have been identified as effective in promoting adaptive changes during therapy sessions are communicated to the family, which is actively involved in collaboratively identifying strategies and tools for implementation in the home environment. AMIRA also conceptualizes motor learning as the outcome of a complex perceptual–motor–cognitive process aimed at solving a meaningful task, emerging from interaction with the environment under conditions that support successful performance [43,44].

Although AMIRA draws inspiration from the above-mentioned established theoretical principles and shares some elements with existing play- and task-based approaches (such as A.M.O.GIOCO) [45], its original contribution lies in the detailed description of a multidimensional early intervention framework specifically tailored for infants at risk or diagnosed with CP, and specifically detailed based on the infant’s motor and sensory limitations. Unlike the majority of studies focused solely on the effectiveness of interventions without providing a detailed description of the underlying methodology, with few notable exceptions [20,46], AMIRA provides a detailed operational guide that defines the goals, activities, adaptations, and therapist–family interaction strategies according to the developmental stages and functional profiles, thus supporting consistent application and standardization among therapists. Furthermore, it integrates the perceptual, motor, cognitive, and emotional domains within a unified, family-centered, and ecologically valid framework. This comprehensive description allows for both replicability in clinical settings and the future evaluation of its effectiveness through controlled studies. As such, AMIRA contributes to bridging the gap between theoretical models and applied rehabilitation practices in early developmental care.

The aim of this article is to present the AMIRA systematic approach, describing the criteria, objectives, tools, and activities outlined in the protocol. A characteristic aspect of AMIRA is that, while it specifies detailed and specific activities for each adaptive function, the proposed interventions are inherently multidimensional and transversal, involving multiple functions simultaneously. These interventions are designed to align with different developmental stages and are graduated in terms of intensity and complexity, embodying the characteristics of an individualized, tailored, intensive, multidimensional, and family-centered approach [28]. In line with this objective, 10 children aged 6 months to 2 years showing signs of a risk or diagnosis of bilateral CP (classified as levels II to IV according to the Gross Motor Function Classification System [GMFCS-ER] [47], Manual Ability Classification System [MACS] [48], and Visual Function Classification System [VFCS] [49]) have been enrolled for the application of the AMIRA approach. These children underwent a 6–8-week treatment program (three sessions per week, each lasting two hours, totaling 48 h) and were compared to 8 children with the same clinical profile (control group) receiving standard care, evaluated using the same protocol. Participants were assessed at baseline (T0), at the end of treatment (T1), and six months post-treatment (T2) using a standardized protocol comprising the following validated scales: Griffiths Scales of Child Development, Third Edition [50]; Gross Motor Function Measure (GMFM) [51]; Peabody Developmental Motor Scale-2 (PDMS-2) [52]; Pediatric Evaluation of Disability Inventory (PEDI) [53]; and Neonatal Assessment Visual European Grid (NAVEG) [54]. A detailed description of the pilot study and its results (founded by the Mariani Foundation) falls beyond the scope of this work and is currently under preparation. In the Results section, a case report is nonetheless provided of an infant who underwent a neurodevelopmental promotion intervention using the AMIRA protocol.

## 2. Materials and Methods

### 2.1. The Methodology and Tools of the AMIRA Approach

**Figure 1:**
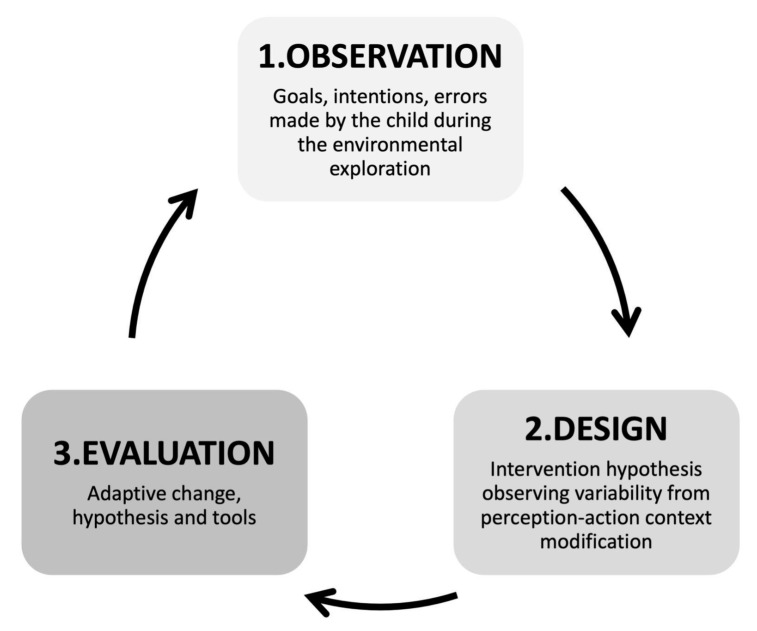
Pierro’s Ecological Rehabilitation [23,24,55].

**Figure 2:**
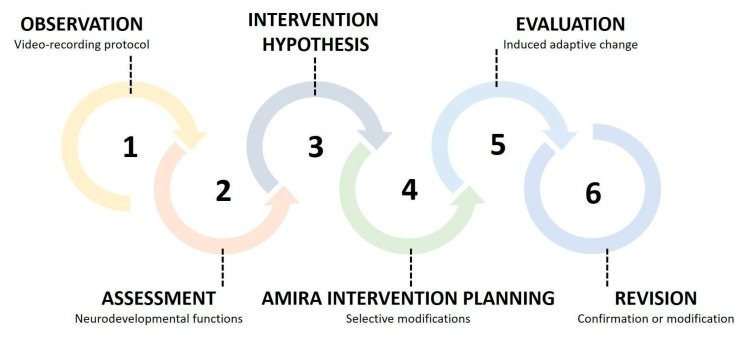
Implementation process for the AMIRA rehabilitation project.

The AMIRA approach is inspired by the Ecological Rehabilitation model developed by M.M. Pierro between 1984 and 1998 [23,24,55] (Figure 1). By introducing the concept of “assessment of adaptive modifiability,” this framework utilizes information gathered during observational and assessment phases to induce an “adaptive change”. This information is then translated into therapeutic activities that facilitate the emergence of new skills transferable to daily life situations. The approach is specifically designed for infants at risk of brain damage and/or neurodevelopmental disabilities within the first two years of life. Although it should be applied flexibly according to the characteristics of both infants and caregivers, as well as the organizational structure of the reference facility, it is recommended that they schedule a minimum of three sessions per week, which may also be delivered via tele-rehabilitation. The duration of each treatment cycle should be determined based on individualized goals. AMIRA is structured to guide early rehabilitation through the following steps: (1) infant observation, (2) assessment of neurodevelopmental functions, (3) development and application of intervention strategies and tools alongside concurrent evaluation of changes induced in the child’s competencies, and (4) assessment of the appropriateness of the strategies employed (Figure 2). In the AMIRA framework, the preliminary evaluation, including eligibility and exclusion criteria, as well as the assessment of neurodevelopmental functions, is conducted by a child neuropsychiatrist with the collaboration of physiatrists and psychologists, ensuring a thorough diagnostic and clinical profile. The infant observation through the video-recording protocol, the intervention hypothesis, and the development of the habilitative treatment are delivered by specialized child therapists, specifically trained in early neurodevelopmental rehabilitation. Beyond individual roles, AMIRA promotes a coordinated interprofessional model involving physicians, therapists, psychologists, and other specialists who work together with the family to ensure a unified and personalized rehabilitation plan. The team is responsible not only for intervention delivery but also for ongoing clinical monitoring and regular interdisciplinary case discussions, ensuring that the approach is comprehensive, specific, timely, and effective, particularly during early developmental windows when neuroplasticity is highest.

*Step 1: The infant observation*

The initial phase is characterized by the observation of the child’s spontaneous behavior to identify needs, intentions, choices, mistakes, and limitations that emerge during the pursuit of personal goals. This observational phase employs the Video Recording Protocol developed by the Italian Cerebral Palsy Group (Gruppo Italiano Paralisi Cerebrale Infantile, GIPCI) [56,57] as a valuable tool for “capturing” elements that can later be re-evaluated and analyzed, facilitating interdisciplinary discussions among professionals. The following key questions should guide the observation: What is the infant attempting to do? What is the infant able to do? What fundamental need is the infant expressing during these attempts?

The focus of AMIRA is to discover and highlight the infant’s needs and goals while safeguarding their emotional, relational, and motivational dimensions through playful interactions mediated by the caregiver. Infants’ emotions, both positive and negative, expressed during spontaneous activities and rehabilitation tasks serve as indicators of motivation and enjoyment, and are essential elements in the decision-making process for selecting therapeutic strategies. Therefore, each intervention should be adjusted based on the continuous monitoring of the child’s reactions, experiences, and behaviors.

*Step 2: The assessment of neurodevelopmental functions*

AMIRA integrates both qualitative multidisciplinary assessment and standardized evaluation protocols. Initially, the infant undergoes a pediatric neuropsychiatric examination, accompanied by instrumental investigations when necessary. Additionally, regulatory, sensory, motor, communicative, and cognitive functions are evaluated using standardized quantitative methods. Key instruments include developmental scales such as the Griffiths III and Bayley III [58], international functional classification systems (e.g., GMFCS, MACS, and VFCS), and adaptive functioning interviews (e.g., Vineland Adaptive Behavior Scales, Second Edition [59]). Once the neurodevelopmental functions targeted for intervention have been identified, concrete short-term intervention goals are established, corresponding to the specific skills to be promoted. In addition to the baseline assessment, the child neuropsychiatrist will conduct a follow-up neurodevelopmental evaluation six months after the end of the treatment, in order to assess the effectiveness of the intervention over time.

*Step 3: Development and application of intervention strategies and tools alongside concurrent evaluation of the changes induced in the child’s competencies*

**Figure 3:**
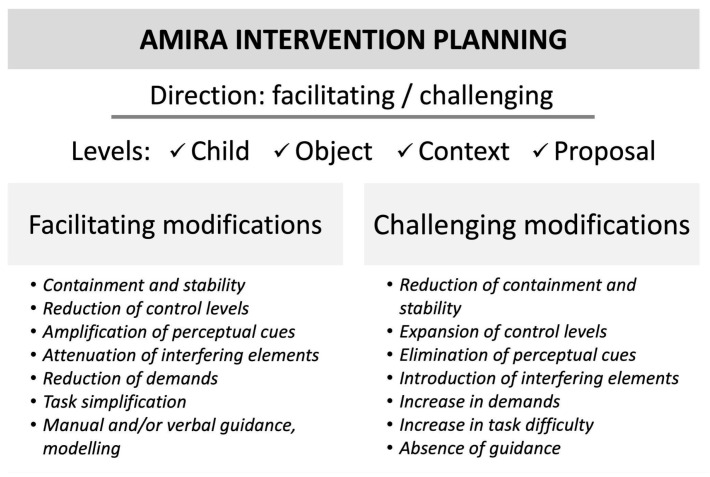
Directions and levels of intervention for modifications.

During the implementation of the activities and through interactions with the child, the therapist observes any behavioral changes elicited by the proposed tasks and by the selective modifications of the context and the objects used. These modifications can be either facilitating (to support the acquisition of new skills) or challenging (to reinforce skills that have already been acquired) (Figure 3). They can be introduced at multiple levels:

- Physical context: Enhancing visual, auditory, and/or verbal cues to guide the child’s attention, exploration, gestures, and movement; creating an environment that facilitates the identification and selection of relevant information.
- Social context: Ensuring the presence of a primary caregiver; fostering a calm and reassuring atmosphere in which both the caregiver and the child feel secure and welcomed.
- Child-specific adaptations: Adjusting the type and degree of physical and tactile support; promoting neurovegetative regulation and postural–motor stability; offering physical and/or verbal cues; modeling the targeted action; when necessary, introducing orthotic devices to reduce the motor control demands associated with the task execution.
- Objects: Modifying objects to ensure they are both perceptually accessible and functionally usable by the child.
- Task/activity structure: Adjusting the level of difficulty according to the optimal challenge level [60], ensuring that the task presents with a reasonable expectation of success for that specific child, at that developmental stage, with those specific difficulties, within that particular life context.

Within the AMIRA framework, postural aids and orthoses are considered when clinically indicated, and are integrated into the intervention as facilitating tools to support functional development. Although AMIRA emphasizes active, goal-directed activities within enriched and motivating contexts, the use of external supports, such as seating systems, standing frames, or orthoses, is fully compatible with its principles when they serve to enhance postural stability and reduce the complexity of motor control demands, thereby facilitating access to more challenging functional tasks, such as fine motor or manipulative activities. These tools are carefully selected based on the individual child’s needs and are introduced in synergy with therapeutic goals, ensuring they promote—not replace—active engagement.

*Step 4: Appropriateness of the strategies employed.*

Following the observational and assessment phases, an intervention hypothesis is established, which is then translated into the identification of goals, the definition of the context and objects, and the related activities, including the calibration of operational choices in a manner that is either facilitating or challenging. Within the framework of ecological rehabilitation [24], a positive change is directly observed by the therapist during sessions. For example, when working with a child who has difficulty with antigravitational head control and visual focus, the therapist might hypothesize that these challenges are linked to visual perception issues. To address this, a high-contrast target is introduced within the child’s visual field to enhance visual stimuli and encourage engagement. The therapist then raises the target above the child’s line of sight, prompting engagement in antigravitational head control. This change is considered successful as it allows the child to explore the environment by integrating visual function with postural control. The emergence of specific actions, abilities, and behaviors that would not have occurred without the targeted intervention underscores the importance of well-formulated hypotheses in addressing the child’s difficulties. If the anticipated change does not occur, the hypothesis is refuted, prompting the therapist to adjust the approach by reformulating the hypothesis and by modifying the context and objects to better align with the child’s needs.

AMIRA intervention begins with a facilitating phase, where the therapist supports the child’s actions before transitioning to active experimentation. Through trial and error, the child explores autonomous strategies, extracting stable action patterns and selecting the most effective ones to achieve desired goals. Key intervention parameters, including environmental characteristics, object selection, type of activity, balance between facilitation and challenge, session duration, and task distribution, must be flexible and tailored to the child’s interests, needs, motivation, and engagement. These adjustments throughout the process help sustain motivation and enjoyment, both across children and within individual sessions.

In line with a holistic perspective of the child as an integrated and dynamic entity interacting with their environment, AMIRA differentiates interventions by function. This differentiation, although not to be understood as mutually exclusive, helps direct attention to a specific skill to be promoted while naturally engaging multiple functions, such as affective, communicative–relational, emotional, perceptual, praxic, postural–motor, and cognitive, within each activity.

The introduction of novel elements during the session is crucial for re-engaging the child’s attention. This can be achieved by alternating or combining objects as needed, in response to fluctuations in attention and engagement. AMIRA respects the child’s exploratory rhythms by incorporating rest pauses and activity transitions. The duration of each specific activity is determined by the child’s progress toward the targeted goal and their sustained interest. A core principle of the intervention, across all functional domains, is the child’s emotional and motivational engagement. Only by respecting their individual needs and rhythms can each activity be meaningfully received and utilized as a learning opportunity.

For each function targeted for intervention, AMIRA outlines the goal (specific skill to be developed), the activity, the contextual characteristics, the child’s positioning during the activity, and the tools or objects to be used.

### 2.2. Facilitating Modifications for Children with Visual Impairment

AMIRA emphasizes facilitating access to materials and fostering play-based interactions between the child, their environment, and communication partners. Particular focus is placed on optimizing both micro- and macro-environments for children with, or at risk of, visual impairment. This approach is cross-functional and, thus, applicable to all the proposed activities. It includes the use of high-contrast visual aids such as checkerboards, striped panels, radial patterns with alternating black-and-white segments, and high-contrast black-and-white images and objects, to enhance visual perception and capture the child’s attention. Checkerboard can be introduced to very young children or those with severe visual impairment. Different high-contrast visual panels serve specific purposes within the AMIRA framework:

- A standalone checkerboard serves as an engaging and perceptually salient target, promoting visual attention, visual–motor skill development, and antigravity head control.
- When used as a background for objects, the checkerboard enhances figure–ground perception, aiding the child in distinguishing object shapes, which is essential for reaching and grasping.
- Striped panels are used to enhance the perception of spatial progression during pre-locomotor and locomotor activities.
- A black-and-white radial pattern on a supporting surface enhances perceptual cues associated with pivoting movements in prone or seated positions.
- A black-and-white radial pattern on a tabletop during object-reaching tasks amplifies spatial displacement cues of the limbs, thereby facilitating visuomotor integration.

Multisensory objects can be also introduced, particularly for individuals with severe visual impairment, to promote the ability to seek and reach objects presented through auditory and tactile cues [61]. The progressive acquisition and mastery of these objects appear to be associated with an increasing awareness of object permanence in the absence of visual input [62,63]. Interaction and communication, like verbal and non-verbal modalities and other supports like storytelling, nursery rhymes, or music, must be used to support weaker areas.

Adapting the environment can further facilitate visual perception and attention. This can be achieved by using soft, indirect lighting to avoid harsh or direct light sources, and by employing a flashlight to illuminate the child’s face, the caregiver’s face, or objects presented during activities. This strategy serves to direct the child’s attention and improve their ability to perceive faces and objects that might otherwise be difficult to detect under natural lighting conditions. For further details, please refer to the Supplementary Materials.

## 3. Results

The AMIRA rehabilitative intervention is an individualized approach that takes into account the specific needs expressed by the child, respecting their exploratory and developmental rhythms. It focuses on what is useful to learn at that moment “for that child, with those difficulties, in that life context, at that stage of development” [24]. The approach follows a gradual progression: an initial observation phase, a “**facilitating**” phase to guide the child’s engagement, and an active experimentation phase where the child independently explores strategies, using trial and error to identify effective methods and extract action rules to achieve goals. Then, the proposal can be gradually modified in a “**challenging**” direction by increasing the complexity and requiring the simultaneous control of multiple functions. The “adapted and tailored” activities allow the child to enjoy the process of acquiring new skills and behavioral strategies that are transferable and reproducible independently and with satisfaction in their daily life context. Successful strategies are shared with the caregivers, who actively participate in the sessions, within a family-centered approach. This enables caregivers to integrate these strategies into daily life, ensuring they can effectively understand and manage the child’s responses, engagement timing, and routines. A core principle of AMIRA is the child’s emotional and motivational involvement (Table S1: Experiences–Emotions–Motivations), as proposals can only be effective learning opportunities when they align with the child’s needs and rhythms. The difficulty level of the proposed activities, whether facilitating or challenging, is carefully calibrated to offer the child a reasonable expectation of success, fostering motivation and encouraging active engagement.

AMIRA categorizes interventions into six functions: behavioral–emotional self-regulation, visual, postural–motor, praxic, interaction–communication, and cognitive functions. For each function, AMIRA charts have been developed with activities of increasing complexity, aligned with the typical developmental milestones up to 24 months of age. However, the reference to these milestones is intended as a guideline rather than a strict framework, as the use of the charts must be tailored to individual needs, particularly in cases involving an older chronological age or more severe clinical impairments. For each specific ability, the habilitative goal, contextual guidelines (including the characteristics of the environment, objects, and tools), and activity proposals are provided. The selection of activities, along with the associated objects and tools, as well as context adaptation, depend on the child’s functional level (also according to the international functional classification systems—VFCS, GMFCS, and MACS), age, and the findings from the assessment phase. The proposals vary in terms of direction (facilitating or challenging), duration, and frequency, depending on the child’s needs, interests, motivation, and behavior observed during the rehabilitative session. The tailored selection and timing of objects and activities presentation, along with periodic novelty, could help to sustain attention.

The Behavioral and Emotional Self-Regulation Function interventions (Table S2: Behavioral and Emotional Self-Regulation Function Chart) comprehensively target key milestones in behavioral and emotional self-regulation and autonomy during early development. These interventions address physiological regulation (e.g., breathing, skin color, rhythms such as sleep/wake, hunger/satiety, and quiet/activity), emotional modulation (e.g., crying, self-soothing, and frustration tolerance), and sensory and social integration (e.g., noise tolerance and shared attention). Autonomy is fostered through goals like independent feeding, object manipulation, and collaboration in daily routines.

The Visual Function interventions (Table S3: Visual Function Chart) are aimed at enhancing core visual and oculomotor abilities, including fixation, smooth pursuit, and goal-directed saccades. These functions are fundamental for the visual exploration of the surroundings and for modulating gestures and movements tuning them to the environment. For this reason, the interventions are implemented in ecologically valid settings. The objective is to foster the development of progressively more functional visually guided actions (functional vision) from the earliest months of life [64,65].

The Postural–Motor Function interventions (Table S4: Postural-Motor Function Chart) target several key abilities: maintaining and controlling various postures in both static and dynamic contexts, including the capacity to respond to external and internal perturbations; adjusting and controlling movements of the head, trunk, and limbs during different activities; achieving autonomous movement on the floor in prone and seated positions, with or without simple assistive devices; transitioning from sitting to standing with or without support, and with or without assistive devices; navigating stairs, overcoming small obstacles, and performing basic functional movements; achieving independent locomotion with direction changes; and transporting objects, such as pushing or pulling toys or using assistive devices or orthotics.

The Manipulative–Praxic Function interventions (Table S5: Manipulative-Praxic Function Chart) focus on enhancing eye–hand and eye–hand–mouth coordination, in order to carry out the following: stabilizing posture to enable effective object grasping and manipulation; adapting grip strategies to diverse objects; facilitating object transfer and visuo-tactile exploration; promoting appropriate object placement based on its properties; and refining anticipatory skills through prehensile experiences; ensuring the practical application and transferability of these abilities to daily life activities.

The Interaction–Communication Function interventions (Table S6: Interaction-Communication Function Chart) are implemented to develop and enhance perception and orientation to sounds, noises, and voices, voice recognition, attention to communication, and intentional and communicative responses, both verbal and non-verbal. The interventions also target socially motivated actions and imitative behaviors, such as waving, pointing, and pretend actions like eating, drinking, or feeding a doll. Additional goals include verbal comprehension of familiar words and everyday phrases, non-verbal communication through facial expressions (e.g., smiling and grimacing) and communicative gestures (e.g., pointing and showing), and verbal communication ranging from crying types, vocalizations, and babbling to simple words and short phrases.

The Cognitive Function interventions (Table S7: Cognitive Function Chart) emphasize the importance of environmental exploration, object manipulation, and social interaction as foundational activities for developing essential cognitive skills such as recognition, memory, and problem-solving. The progression from simple to more complex activities is designed to foster flexible thinking, while the focus on spatial and causal relationships with objects supports cognitive adaptability and analytical skills. The interventions also focus on attention, memory, and self-determination, empowering children to strengthen their cognitive capacities through active engagement, imitation, and choice-making. This chart has been constructed following an evolutionary sequence of learning steps aligned with the child’s developmental progression. Specific goals and therapeutic proposals were derived by standardized developmental scales [50,58], from which various items were extracted and integrated to define a correct progression of cognitive skills.

See Table 1 for an example of a multidimensional and integrated proposed activity.

### Case Report

M. is the first-born of a monochorionic diamniotic twin pregnancy, which was uneventful until the 30th week of gestation, when increased uterine resistance and intrauterine growth restriction were detected. She was delivered via cesarean section at 30 + 6 weeks due to a non-reassuring fetal heart rate (Apgar score: 5/9; birth weight: 1495 g). A brain MRI at a term-equivalent age revealed bilateral fronto-parietal cystic periventricular leukomalacia, associated with supratentorial ex vacuo ventriculomegaly and marked hypoplasia of the corpus callosum. After being discharged from the Neonatal Intensive Care Unit, M. began neurodevelopmental follow-up at the Unit of Child Neurology and Psychiatry, ASST Spedali Civili of Brescia, Italy, and received standard habilitation care until 12 months corrected age, when she was enrolled in the AMIRA pilot study and began the intensive intervention protocol (2 h sessions, three times per week for 8 weeks).

Her clinical profile included bilateral motor impairment, predominantly affecting the lower limbs and the right upper limb. Functional classifications were as follows: GMFCS level IV, mini-MACS level III, and VFCS level II. The Griffiths III developmental profile showed significant discrepancies, with deficits in the gross motor (developmental quotient, DQ: 62) and visuomotor coordination (DQ 76) domains, contrasted by relative strengths in language (DQ 88), learning foundations (DQ 84), and socio-emotional (92) areas.

Based on the assessment, an individualized rehabilitation plan was designed, targeting the following domains: emotional and motivational regulation (e.g., frustration tolerance), visual function (e.g., saccadic eye movements), gross motor skills (e.g., achieving sitting position), fine motor and praxis abilities (e.g., eye–hand coordination and bimanual integration), communication and interaction (e.g., receptive and expressive vocabulary), and cognitive functions (e.g., object permanence and spatial relations). After analyzing M.’s needs and motivations, gross motor and manipulative–praxis functions were identified as crucial areas to be supported through facilitating proposals. A quiet environment was created both physically and relationally (i.e., a relaxed and reassuring atmosphere), and an affectively significant figure was included in the setting to help soothe the child. To promote postural control, for example, the child was positioned sitting on the mat with support to the lower trunk, and balancing activities were proposed along with the elicitation of parachute reflexes. Motivation was sustained through the use of rhythmic songs and nursery rhymes. To foster hand–eye coordination, facilitating strategies were again employed: the child was seated on a small bench with trunk support and offered an attractive and easily graspable object. In a subsequent phase, to encourage adaptability of grasp, objects of varying shapes, textures, and sizes were introduced. Throughout the activities, the child was verbally encouraged and physically guided in her movements when necessary.

At the six-month follow-up, M. showed overall developmental progress, with notable gains in communication, social engagement, and emotional regulation (communication DQ 99, and socio-emotional area DQ 98). The improvements in the gross motor and visuomotor areas were less pronounced possibly due to the sensitivity of the standardized tools. These gains were better captured through video analysis, therapist observation, and parental feedback. The high level of family and school collaboration allowed for the transfer of habilitative strategies beyond the therapy setting. Continuous dialogue with the family enabled the team to tailor the intervention to M.’s needs and empowered the caregivers to actively support the therapeutic process.

## 4. Discussion

In this study, the theoretical framework and structure of the AMIRA approach are delineated by formalizing its criteria, objectives, tools, and intervention procedures. This rehabilitative approach has been defined and refined over decades within the framework of the Italian Cerebral Palsy Group (Gruppo Italiano Paralisi Cerebrale Infantile, GIPCI, sustained by Mariani Foundation) which, since 2000, has organized training courses and meetings to discuss theoretical models for the development of rehabilitative approaches for infants and children with CP, based on the principles of early intervention [28,35,66]. Although AMIRA is specifically designed for children with CP, our clinical practice suggests the possibility of its application to be beneficial in other neurodevelopmental disabilities and complex disabilities. The present approach contributes to the provision of detailed and systematized content for defining and planning early intervention domains—encompassing the motor, sensory, cognitive, communicative, and behavioral aspects in a comprehensive approach to neurodevelopment. This theoretical framework aims to integrate all the essential elements of a successful early intervention, which is characterized by being tailored to each infant, multi-axial, family-centered, incrementally challenging, and intensive [7,28], while also incorporating a structured and controlled design to enhance reproducibility. This framework is designed to guide clinical–rehabilitation reasoning and does not aim to prescribe a rigid sequence of activities to be presented in a predetermined order to the child undergoing the intervention. The suggested activities outlined in the protocol and described in the results should be applied in clinical practice with flexibility and creativity by practitioners and shared to family members supervised by the clinical team in order to be generalized in daily life. The tables presented in the Supplementary Materials section should not be considered as a list of activities but rather as a guide—a framework for examples—that can never be exhaustive in relation to the complexity of the rehabilitation process. The activities are interrelated, and the division into tables is solely for instructional and illustrative purposes. It is not the intended method of use, as the professional is expected to consult not just a single table, but all of them collectively. Both in routine clinical practice and in the implementation of intervention studies, it is essential that we establish precise monitoring measures to evaluate treatment progress alongside the definition of the intervention program. This is achieved through the use of international scales during the initial clinical assessment, before the intervention, and a subsequent post-intervention evaluation to assess the outcome. This requirement also applies to the implementation of the AMIRA intervention. Appropriate outcome measures have not been fully detailed in this study, as they must be defined based on the specific treatment objectives, the chronological or developmental age of the infant or child involved, and the availability of standardized assessment tools in the country of residence. In addition to psychometric evaluations conducted directly with the child during clinical monitoring, it is recommended that we involve both primary and secondary caregivers who are actively engaged in the child’s daily care, through specific interviews or questionnaires, that should ideally address both the micro-objectives targeted in the intervention—thus being ad hoc designed for the treatment—as well as incorporate standardized and validated assessment tools.

Finally, the AMIRA approach aligns closely with the “F-words” framework proposed by Rosenbaum and Gorter (2012) [67], which advocates for a paradigm shift from a deficit-focused model to a strengths-based, family-centered perspective grounded in the ICF. Each of the six F-words—Function, Family, Fitness, Fun, Friends, and Future—is reflected in the structure and rationale of AMIRA. Function is addressed by focusing on meaningful, goal-directed activities that promote participation in daily routines, rather than merely improving isolated motor skills. Family plays a central role throughout the intervention process: caregivers are actively involved in assessment, goal-setting, and implementation, and are supported in transferring strategies to the home environment. Fitness is promoted through intensive, personalized activities that support both motor and cognitive–emotional well-being. The dimension of Fun is embedded in AMIRA’s use of engaging, motivating contexts that foster exploration, learning, and intrinsic motivation. Friends are considered in relation to the child’s emerging social participation, especially in early contexts such as family, nursery, and peer interactions. Finally, AMIRA is inherently oriented toward the Future, with individualized goals that respect the child’s developmental trajectory and potential, aiming to build competencies that support long-term adaptation and quality of life. In this way, AMIRA operationalizes the F-words in a concrete clinical context, translating the ICF into daily rehabilitation practice.

### AMIRA Approach: Limitations and Challenges

This protocol addresses the significant challenge of presenting a wide range of function-specific activities within an integrated perspective. The proposed list of activities should not be viewed as merely sequential, as the implementation of exercises aimed at enhancing a particular function may also indirectly support and enrich other functional domains.

Several limitations and challenges of the protocol can be identified based on the clinical experience gained over years of its application. One critical issue has emerged in the implementation of the protocol, particularly concerning the intensive treatment approach, which is primarily offered in an outpatient setting. This requires a significant effort from families, who often need to undergo a substantial reorganization of their family setting to adhere to the study. Such reorganization is not always easy to manage and may involve other caregivers of the child, such as grandparents, relatives, or babysitters. In past experiences, for families able to sustain the effort required by the study protocol, the process was perceived as a highly productive period for their child’s development, with their efforts being compensated by observed improvements. Given the necessary expansion of the participant pool and the interactions among various stakeholders (parents, grandparents, and other caregivers), it is crucial that we establish dedicated spaces for periodic meetings and discussions with families. Additionally, remote connections could be implemented to monitor the adherence to the program. A key challenge could be the transfer of the habilitative pathway into home and/or school settings through telemedicine monitoring, better addressing family needs, as already experimented in the treatment of other neurodevelopmental disabilities [16]. During the post-intensive rehabilitation phase, families may experience a sense of “abandonment.” Therefore, it would be beneficial to introduce a post-intensive phase that supports families during the transition from an intensive approach to one with a lower treatment frequency or between different hospital and community-based rehabilitation facilities.

## 5. Conclusions

In conclusion, this study provides, like few others available [45], a detailed intervention approach, sharing with the scientific community both the framework of application and a well-structured set of activities that can be implemented in any rehabilitative setting. The feasibility of these rehabilitative contents extends beyond infants and children at risk of neurodevelopmental disabilities or those with CP; more broadly, they can also be applied to various neurodevelopmental disabilities, whose boundaries remain fluid and challenging to define, especially in the first years of life [11,68]. The hope of this study is to foster increased sharing within the scientific community, not only of the theoretical and methodological frameworks of early intervention programs but also of their concrete contents, encouraging a more extensive debate on the topic.

## Figures and Tables

**Table 1:** Example of a multidimensional and integrated proposed activity.

Identification of the “critical” function	Postural control
Identification of the child’s need	Need for stability and security
Facilitating proposal	Activity performed on the caregiver’s lap, who sings a familiar nursery rhyme while supporting the child at the pelvis
Induction of variability with facilitating intervention	The caregiver’s stabilization of the pelvis allows for trunk control and tolerance of balance disturbances
Identification of specific skill (challenging proposal with function integration)	Activation and training of trunk balance reactions during self-induced perturbations, as the child moves the upper limbs to reach and grasp an object of interest
Facilitating perturbation dosage	Pelvic stabilization enables the child to actively control the trunk, while adjusting the distance and direction in reaching the object of interest allows for control over self-induced balance disturbances
Definition of contextual characteristics	Presence of the caregiver; calm environment; absence of disturbing or distracting elements; soft lighting in the room; flashlight beam directed at the object to be grasped if there are visual perception difficulties
Definition of object characteristics	A motivationally interesting toy; small enough to be grasped with one hand (soft and/or small in size); with high chromatic contrast and/or illuminated from within or by the flashlight beam if there are visual perception difficulties
Definition of challenging direction in the proposal	The caregiver sings a song while presenting the object in an area that is easily reachable by the child with slight trunk imbalance. Gradually, the child’s upper limb action range is expanded during the reaching activity. The object is progressively moved to different spatial positions (anterior, anterosuperior, anteroinferior, right and left lateral superior and inferior, and posterior inferior)
Challenging sequence of the proposal	Reducing pelvic support; shifting the proposal from the caregiver’s lap to a flat surface; expanding the space for reaching the object; reducing perceptual facilitations in the context and/or object; moving the object of interest into peripersonal spaces, requiring the activation of support reactions, parachute responses, balancing reactions, and recovery of trunk verticality
Facilitating sequence for the activation of support and parachute reactions	Use of a checkerboard on the supporting surface on which the object of interest is placed.

## Data Availability

Data supporting the conclusions of this article will be made available by the authors, without undue reservation.

## Supplementary Materials

The following supporting information can be downloaded at: https://www.mdpi.com/article/10.3390/children12081003/s1, Table S1: Experiences–Emotions–Motivations; Table S2: Behavioral and Emotional Self-Regulation Function Chart; Table S3: Visual Function Chart; Table S4: Postural-Motor Function Chart; Table S5: Manipulative-Praxic Function Chart; Table S6: Interaction-Communication Function Chart; Table S7: Cognitive Function Chart.

## Abbreviations

The following abbreviations are used in this manuscript:

AMIRA	A Multidimensional and Integrated Rehabilitation Approach
CP	Cerebral palsy
CVI	Cerebral Visual Impairment
GIPCI	Gruppo Italiano Paralisi Cerebrale Infantile
GMFCS	Gross Motor Function Classification System
MACS	Manual Ability Classification System
SIMFER	Società Italiana di Medicina fisica e riabilitativa
SINPIA	Società Italiana di Neuropsichiatria dell’Infanzia e dell’Adolescenza
VFCS	Visual Function Classification System
