# Supplementary material for: A Multidimensional and Integrated Rehabilitation Approach (A.M.I.R.A.) for Infants at Risk of Cerebral Palsy and Other Neurodevelopmental Disabilities
Source: Children (Basel). 2025 Jul 30;12(8):1003. doi: 10.3390/children12081003 (PMC12384761; doi:10.3390/children12081003)
Supplement: Supplementary file 1 [file children-12-01003-s001.zip › Table S1 - Experiences – Emotions – Motivations.pdf]

Table S1 - Experiences – Emotions – Motivations

These principles are universally applicable across rehabilitative contexts and essential for successful interventions. Observing the child's behaviors and responses ensures that activities align with their needs, motivations, capabilities, and readiness. The level of difficulty of the various proposals (chosen in a facilitating or challenging direction) must take into account the respect for the optimal challenge level, that is, the level of difficulty that allows the child a reasonable expectation of success and motivates them to take action.

Function	Objective	Dysfunctional Parameters	Proposals
Need for stability	Self-stabilization abilities via bracing or postural-motor organization along the midline.	Any variation in position or environment disrupts the child, causing frequent startle reactions, increased irritability, unwillingness to engage with proposals, frustration, and crying.	• Create a welcoming and stable environment.<br>• Establish reassuring and reliable relationships.<br>• Ensure a comfortable, stable, and secure position for the child.<br>• Propose tasks and activities in a consistent and routine manner.<br>• Create stable interaction situations, introducing small variations gradually, adjusting quality and quantity according to the child's reactions.<br>• Avoid sudden changes in position, support, or proposals.<br>• Prevent disruptive elements (e.g., noises, voices, external movements).<br>• Continuously monitor the child’s level of calmness.<br>• Ensure reciprocal exchange between parent and practitioner to transfer proposals into the child's everyday context.
Need for security and protection	Ability to adapt to environmental changes and therapeutic procedures.	The loss of physical contact or containment results in increased tension, fear, and behavioral instability.	• Create a calm environment.<br>• Foster a relaxed and welcoming relational climate.<br>• Provide physical containment for the child.<br>• Verbally reassure both the child and parents regarding proposed activities, the child's responses, and address any concerns or fears.<br>• Include an emotionally significant figure in the setting to comfort the child.<br>• Integrate emotionally meaningful objects into the setting.<br>• Adjust proximity and distance appropriately.<br>• Present tasks and activities in a stable and routine manner.<br>• Establish stable interaction situations, introducing small variations gradually, adjusting quality and quantity based on the child's reactions.<br>• Continuously monitor the child’s level of calmness.

Need for exploration and knowledge	Curiosity and interest towards contextual information with activation of attentional orienting behaviors.	The child does not show interest or curiosity toward environmental information, appears indifferent or unavailable, avoids exploring the space, appears frightened, hesitant, and resistant to prompts; if moving, does so within a very restricted area.	• Identify perceptually relevant facilitations for the child to aid in figure/ground perception and to define spaces for action, movement, and locomotion.<br>• Amplify perceptual cues related to distances, orientations, directions, and positions to enable clear detection or access to spatial cues within the environment.<br>• Set up the context with facilitations to help the child identify and define spaces for action, movement, and locomotion.<br>• Allow the child to actively explore distances, orientations, directions, and positions of objects, environments, and people.<br>• Break down and verbalize the topological and metric aspects of actions, movements, and locomotion during their execution.<br>• Facilitate active exploration of action rules.
Need for encouragement and esteem	Tolerance for frustration in delayed achievement of goals and maintaining high motivation for success.	Tends to lose motivation and, due to low confidence in their abilities, constantly requires the presence, guidance, and support of an adult.	• Verbally encourage and support the child throughout the activity.<br>• Adjust demands based on the child’s actual capacity for understanding and action.<br>• Propose tasks that allow for gratification and success through a proper definition of the child’s "optimal challenge level”.<br>• Teach the child to tolerate waiting and small frustrations resulting from delayed gratification of requests and desires, using the “counting game” approach: “Wait, I’ll count to 3 and then I’ll give it to you…” and progressively help the child tolerate longer waiting times.<br>• Demonstrate the action calmly to the child multiple times, describing it verbally.<br>• Physically guide the child's movements and actions if necessary.<br>• Celebrate the child's success with enthusiasm.<br>• Discuss failures with neutral, encouraging tones.
Need for challenge and success	Pleasure in testing oneself by facing new or challenging situations beyond acquired and consolidated abilities.	Avoids testing oneself to prevent failure and frustration.	• Design simplified activities based on the "errorless learning" principle.<br>• Identify the child’s actual skill level.<br>• Allow practice until behavioral mastery of the skill is achieved.<br>• Define the next level of difficulty that provides the child with a reasonable expectation of success.

			• Adjust the amount and number of challenges for each task.<br>• Propose problems that the child can solve independently, with minimal or no assistance.<br>• Encourage the child to find their own solutions for small problems in daily activities.<br>• Allow space for active experimentation by the child.<br>• Permit mistakes and encourage retrying.<br>• Guide the child in analyzing errors.<br>• Assist in identifying more effective strategies if necessary.<br>• Celebrate their success enthusiastically.
Need for mastery	Engage in maintaining attention on a single activity with a high level of motivation until it is mastered effectively.	Due to the tendency to avoid the "frustration of failure," the child avoids taking initiative and engaging in activities or practicing to gain mastery.	• Present the child with adaptive tasks and solvable problems within their reach, allowing them to experience success.<br>• Design simplified activities based on the "errorless learning" principle.<br>• Allow the child to make repeated active attempts to solve tasks until mastery is achieved.<br>• Respect the time needed for the child to produce these attempts.<br>• Respect the strategies the child identifies and adopts if they prove functional and adaptive.<br>• Enable the child to find their own solutions to small problems in daily activities.<br>• Guide the child toward alternative problem-solving strategies that can be promptly used.<br>• Allow the child to experiment as the author of their own actions.<br>• Permit the child to "try and retry," gaining practice with the learned strategy, even in various real-life contexts.
Need for fun and shared play	Engage with pleasure during activities and remain open to social interaction for a sufficient period to complete a short shared game.	Shows little interest in playful interaction and game proposals, tending to retreat into repetitive, self-soothing, and self-stimulatory activities.	• Create a shared atmosphere of play and enjoyment, considering the child's preferences and attentional and interactive rhythms.<br>• Set up body-based and interactive play situations that elicit clear signs of pleasure and enjoyment, pausing them and waiting for a request (vocalization, eye contact, gesture) to resume the game sequence.<br>• Use nursery rhymes and songs to accompany movements that may destabilize or scare the child.

			• Choose objects and toys together with the child.<br>• Set roles, turns, scores, and outcomes.<br>• Agree on the game's duration and ending.<br>• Establish new rules if needed.<br>• Be prepared to change games when the child shows disinterest.
