# Supplementary material for: A Multidimensional and Integrated Rehabilitation Approach (A.M.I.R.A.) for Infants at Risk of Cerebral Palsy and Other Neurodevelopmental Disabilities
Source: Children (Basel). 2025 Jul 30;12(8):1003. doi: 10.3390/children12081003 (PMC12384761; doi:10.3390/children12081003)
Supplement: Supplementary file 1 [file children-12-01003-s001.zip › Table S2 - Behavioral and Emotional Self-Regulation Function Chart.pdf]

Table S2 - Behavioral and Emotional Self-Regulation Function Chart

Premises for using the chart

- The contents of this area should be applied transversally to all the rehabilitative contexts indicated for each specific function and are considered fundamental both for the child's acceptance of the proposals and for the success of the rehabilitation intervention itself. In order to accept a proposal, it is necessary that the person making the proposal tunes in to the child's emotional state, respects them, and knows how to interpret their emotional, tonic, motor, expressive, and verbal reactions. To tune in to the needs, motivations, and experiences of the child, it is important to observe every behavioral manifestation during the interaction. This attention allows the offering of proposals that respect the child's possibilities and availability. The level of difficulty of the different proposals (chosen in a facilitating or challenging direction) must respect the child's "window of tolerance" to the stimuli induced by the parent, caregiver, or therapist. Attention and respect for the child's tolerance limits and safeguarding their well-being allow for dosing the optimal challenge level in each proposal, that is, the level of difficulty that protects the child from stress, frustration, and rejection, providing them with a reasonable expectation of success that motivates them to act.
- All the proposals described below refer to a rehabilitative approach that considers the child in its entirety, that is, as a mind-body unit. According to this perspective, all the functions are closely connected to each other and are organized each time, cooperating to achieve a specific goal for optimal adaptation of the child to the surrounding environment. If cooperation between multiple functions is not possible or is difficult, and thus optimal adaptation of the child to the living environment cannot be achieved, the characteristics of the environment must be adapted to the child's needs and requirements through perceptual-motor facilitation interventions.
- Proposals that are effective in producing an adaptive change in the child during therapy should be shared with the family, working together to find strategies for transferring them to the home environment. Family members should be supported in understanding the goals of the various proposals, in paying attention to the child's reactions, and in managing the timing of the proposals (e.g., when during the day, in which daily life situations).
- The selection of objects and activities, as well as the adaptation of the context (from the options indicated in the table), are variable and depend on the child's functional level, following the indications provided by the classification scales (VFCS; GMFCS, Mini-MACS). The choice of the direction of the proposal, whether facilitative or challenging, the duration and number of proposals, and the time to be dedicated to each individual proposal within the rehabilitative plan must necessarily vary from child to child and, for the same child, from session to session, depending on their interest, needs, motivation levels, and availability, in order to support their motivation and enjoyment of learning.
- In the presence of visual engagement difficulties, it is recommended to evaluate the opportunity of using a chessboard and/or high-contrast black-and-white images and objects that can amplify the visual perception cues related to the objects in use and the child's action context. These precautions help facilitate the child's attentional

orientation, enabling the integration of information from the visual channel with the other functions. The chessboard can be used alone as an attentional cue or as a background to objects, amplifying the perception between objects and background. Another useful precaution is to provide soft lighting in the room (free from direct and intense light sources) and use a flashlight to illuminate the child's or caregiver's face, or the objects being proposed.

- If a decline in attention and availability is observed, it is useful to introduce novel elements to regain their attention. This can be achieved by alternating the use of objects (from those described) or using them in combination (e.g., face + flashlight; rattle + chessboard + flashlight, and so on).
- It is useful to schedule rest breaks and change activities when the child shows no further interest in the ongoing activity.
- The overall duration of the proposed activity is related to the child's achievement of the objective and their motivation to continue pursuing it.
- The age division is indicative, and it is possible, for each age group, to introduce activities and objects described in previous age groups.

Objectives for Behavioral and Emotional Self-Regulation Function

- Adaptation to the extra-uterine environment: regulation of breathing, color, rhythms: sleep/wake, hunger/satiety, calm/activity
- Cry modulation and self-soothing
- Sucking, chewing/swallowing, duration of the meal, posture during the meal
- Reaction and tolerance to noises
- Shared attention
- Regulation of muscle tone
- Active participation in social interaction
- Tolerance of frustration and waiting
- Ability to differentiate between familiar and unfamiliar figures and overcoming fear of strangers
- Evolution of the attachment-separation process and initial experimentation of psychological autonomy from reference figures
- Autonomous exploration of the environment supervised by the adult
- Autonomy in self-feeding with hands
- Ability to use a utensil roughly
- Ability to open a box to extract its contents
- Tolerance/collaboration during dressing, undressing, and washing activities

Age appropriate tools

**0-6 months**	**6-12 months**	**12-24 months**	**Contextual elements**
Human face, pacifier, baby bottle, nipple shields	Human face	Human face	Human face
Flashlight	Flashlight	Building blocks	Room lighting, flashlight
Checkerboard	Checkerboard	Everyday objects (plate, spoon, fork, cup, small pot, brush)	Emotionally significant family member
Fantz face	Multimodal toys	Books with flaps	Mother's arms, DouDou (comfort object)
Bull's-eye	Containers	Animal figures	Reclining seat, infant car seat, highchair, nursing pillow, stroller, soft containment rolls, donut-shaped pillow, cradle
Necklaces	Rattles	Toy cars	Wedge, roller
Graspable ball	Soft books	Pull-back toy cars	Soft step structure
Soft ball	Push-button toys	Puzzles with increasing complexity	Cube or table (40 cm in height)
Sensory ball	Spinning top	Paper and markers	Checkerboard
Bells	Sound-producing toys	Velcro fruit toys	Rocking platform
Spring toy	Graspable objects and toys	Rings	Sensory surfaces
Koosh ball	Bimanually articulated objects	Pull-along toy with a string	Black-and-white striped surface
Sound-emitting ring	Musical instruments	Images and photographs of	Musical mobile

	(keyboard, drum, maracas, rattles, rain stick)	everyday objects	
Ribbons with bells	Napkin for object hiding games	Small roller, 15 cm in diameter	Posture support system
Nursery rhymes and songs	Nursery rhymes and songs	Nursery rhymes and songs	Music, nursery rhymes and songs

Behavioral and Emotional Self Regulation Function chart

0-3 months						
**Ability**	**Objective**	**Context**	**Child**		**Tools**	**Proposals**
**Regulation of breathing, complexion, and sleep**	Promoting a regular sleep-wake rhythm.	Everyday living environment (home).	Deep sleep	Eyes closed, absence of eye movements, regular breathing. Relaxed muscle tone, absence of motor activity.	Stroller.<br>Crib.<br>Mother's arms.<br>Bouncer/Car seat.<br>DouDou.	Supine position in the crib.<br>Supine/prone position in mother's arms.<br>Skin-to-skin contact.<br>Non-nutritive sucking.<br>White noise.<br>Low-light environment (soft lighting).<br>DouDou placed near the baby.
			Light sleep	Fixed pupils with rapid eye movements, regular breathing. Some movements in the face and limbs.		
			Drowsiness	Half-closed pupils. If slightly open, the eyes do not focus on anything. Breathing is fairly regular. Presence of some movements.		

			Alertness	Eyes open. The baby is calm and attentive to the environment (especially to the mother when she looks at them). Regular breathing.		
			Agitation	Facial grimaces present. Irregular breathing. Skin intermittently flushed.		
**Modulation of crying and self-soothing**	Consolability.	Everyday living environment (home).	Screaming and crying. Grimaces, widespread motor activity, contractions, red complexion, irregular breathing.		Use of lullabies.<br>Pacifier.	Kangaroo Therapy.<br>Environmental adjustments.<br>Skin-to-skin contact.
**Sucking (rhythm and mouth pressure) and feeding**	Promoting sucking for nourishment and consolability.	Everyday living environment (home).	Sucking	Mouth not latched onto the breast: Anterior-posterior movement, hypotonic (milk loss from the mouth with air swallowing).	Breastfeeding pillow.<br>Bottle.<br>Nipple shields.	Supine position (or with minimal inclination) in contact with the mother. Nipple shields to support sucking.<br><br>Kangaroo Therapy, Wrapping, Nesting to promote the positioning of the upper limbs in the midline and the proximity of the hands to the mouth for self-soothing.
				Tonic anterior-posterior movement.		
**Shared attention***	Promoting attentional reorientation.	Everyday living environment (home).	Social visual targets	Attentional disengagement (shifting attention from social visual targets to objects or toys).	Sound objects.<br>High-contrast objects.<br>Graspable objects.<br>Play mat.<br>Rolled-up towels or rollers.	The parent directs the child's attention (while the child is being held, lying supine on the play mat, or semi-sitting in the car seat or bouncer) to people present in the environment or to new multisensory objects and toys (visual,

					Bouncer.<br>Car seat.	auditory, tactile).
**Regulation of muscle tone**	Supporting the modifiability of motor patterns.	Everyday living environment (home).	Normality of motor patterns.	Spontaneous motor activity characterized by richness, variability, and fluency.<br>Prevalence of flexor patterns and modifiability of motor schemas.	Cradle.<br>Reclining seat.<br>Infant car seat.<br>Broad and comfortable support surfaces.<br>Caregiver's arms.	Situations that vary throughout the day, promoting the child's ability to adapt to different conditions and positions while closely monitoring their reactions to changes.<br>Supporting the child in holding their head when they are unable to do so independently.
	Promoting antigravitational muscle tone and limb midline alignment.	Everyday living environment (home).	Hypotonia	Upper limbs distant from the midline, lower limbs externally rotated and slightly flexed, resting on the support surface (the "frog" position).	Reclining seat.<br>Infant car seat.<br>Postural aids.<br>Rolled towels or small rolls.<br>Cushions.<br>Caregiver's arms.	Facilitate the expression of global spontaneous motor activity in compact positions.<br>Vary the child's positions to promote adaptability and motor exploration.<br>Provide head support when the child is unable to hold it independently.
	Promoting fluid muscle tone and limb midline alignment.	Everyday living environment (home).	Hypertonia	Asymmetric positions characterized by the dominance of the asymmetric tonic neck reflex ("fencer's posture"), spinal hyperextension, and a prevalence of global patterns (simultaneous extension and flexion of the limbs).	Reclining seat.<br>Infant car seat.<br>Postural aids.<br>Rolled towels or small rolls.<br>Cushions.<br>Caregiver's arms.	Compact Position in Caregiver's Arms<br>Encourage compact, midline-aligned positions with flexed limbs using various supportive materials.<br>Avoid abrupt movements to prevent startle reactions, which may lead to postural-motor instability and motor disorganization.<br>Support the child's head and gently realign it with the trunk if hyperextension occurs.

	Promoting stable muscle tone and limb midline alignment.	Everyday living environment (home).	Tone fluctuations	Alternation between hypotonic and hypertonic states.	Reclining seat.<br>Postural aids.<br>Rolled towels or small rolls.<br>Cushions.<br>Caregiver's arms.	Compact position in arms or using supportive materials.<br>Facilitate compact positions using various materials to support postural alignment.<br>Avoid abrupt movements to prevent postural-motor instability, startle reactions, and motor disorganization.<br>Provide head support and gently realign it with the trunk if hyperextension occurs.
	Promoting stable muscle tone and limb midline alignment.	Everyday living environment (home).	Spasms and dystonia.	Presence of involuntary movements associated with phases of hypertonia.	Donut-shaped cushion.<br>Rolled towels or small rolls.	Encouraging compact positions to reduce spasms and dystonia while promoting functional motor acts.<br>Avoid abrupt movements to prevent postural-motor instability, startle reactions, and disorganization of movements and posture.<br>Provide head support and gently realign it with the trunk if hyperextension occurs.
**6-12 months**						
**Ability**	**Objective**	**Context**	**Child**		**Tools**	**Proposals**
**Active participation in social interaction****	Facilitating shared attention.	Everyday living environment (home, nursery).<br><br>High chair.<br>Postural support system.<br>Mat.<br>Small table.	Difficulty with shared attention	The caregiver can capture the child's attention by directing it toward themselves or a motivating multisensory toy, in order to reorient the child's focus and facilitate interaction.	Multisensory games involving vision, hearing, and touch.<br><br>Songs and nursery rhymes.<br>Bubbles, balloons, musical instruments.<br>Tickle games, peek-a-boo game.	In situations of shared pleasure and enjoyment, the caregiver engages the child in socio-sensory play routines, with and without objects.<br><br>The child, either in a supine or seated position, and the caregiver in a frontal position, while encouraging moments of attention triangulation between themselves and an object.

Frustration tolerance**<br>**Attachment-separation**	Promoting secure attachment and a calm separation from the attachment figure.		Starting from 8 months, the child experiences frustration when the attachment figure is absent.	Caregiver.<br>Strangers (nursery teacher/relatives).	Facilitating proposals: the mother maintains physical proximity to the child to provide security while an unfamiliar person enters the situation.<br>Challenging proposals: gradual exposure to the mother's absence, with the involvement of other people who pleasantly distract the child.<br>It is helpful not to immediately respond to the child's crying, picking them up only after using voice and tactile contact for comforting and reassurance.<br>Facilitating proposals: offer non-nutritive sucking (pacifier/fingers in mouth) to encourage self-soothing.
Stranger anxiety**			From 9-10 months, the child distinguishes the mother from a stranger, displaying mistrust toward the latter.		Facilitating Proposals: the mother maintains physical proximity to the child to provide security.<br>Challenging proposals: gradual exposure to the mother's absence, with the involvement of other people who pleasantly distract the child.
			**12-18 months**		
**Ability**	**Objective**	**Context**	**Child**	**Tools**	**Proposals**
Frustration tolerance**	Promoting a regulated response to frustration.	Everyday living environment (home, nursery).	Understands the 'no' as a sign of maternal disapproval: the child begins to differentiate between what is prohibited and what is allowed by the caregiver, inhibiting behavior before it is enacted.	Visual support (gestures and images).	Provide the child with a few clear rules using simple language to delineate and clarify what they can and cannot do.<br>Gradually increase the child’s tolerance for waiting times in meeting needs and requests, through structured pacing and practice.<br>Recognize the child’s ability to adapt to

					waiting by praising and rewarding them with social approval and rewards (hugs, cuddles, etc.).
**Experimentation***			Mother as a secure base for exploration.	Materials for sensorimotor play: cause-and-effect toys (puzzles, building blocks, shape sorter, books, sound toys, spinning top, etc.).<br>Mat.<br>Small table.	Enrichment of the environment with motivating and engaging objects and toys. Sensitivity and receptiveness to emotional experiences of anxiety and fear, with prompt availability for comfort by the primary caregiver.
**Child's autonomous exploration supervised by the caregiver****			The child actively explores the environment while still needing the presence of the mother.		
**Frustration tolerance****			The child is able to modify and regulate their behavior according to the demands of the context and act in accordance with environmental expectations.	Tangible social reinforcements: approval, cuddles, and positive attention from the caregiver toward the child.	In case of failure, support the child to prevent loss of motivation and introduce possible task facilitations so that they can experience success.
**Child's autonomous exploration supervised by the caregiver****			Control is provided by the development of behavioral inhibition mechanisms and the internalization of social and parental prohibitions (from 24 months onward).	Materials for sensorimotor play: cause-and-effect toys (puzzles, building blocks, shape sorter, books, sound toys, spinning top, etc.).	Anticipate the child's understanding of the temporal and/or sequential structure of activities and events. Provide the child with emotional and physical support (containment). Maintain a consistent system of rules in the child's living environments.

* Please refer also to the Cognitive Function chart.

** Please refer also to the Interaction-Communication Function chart.
