# Supplementary material for: A Multidimensional and Integrated Rehabilitation Approach (A.M.I.R.A.) for Infants at Risk of Cerebral Palsy and Other Neurodevelopmental Disabilities
Source: Children (Basel). 2025 Jul 30;12(8):1003. doi: 10.3390/children12081003 (PMC12384761; doi:10.3390/children12081003)
Supplement: Supplementary file 1 [file children-12-01003-s001.zip › Table S3 - Visual Function Chart.pdf]

Table S3 - Visual Function Chart

Premises for using the chart

- All the proposals described below refer to a rehabilitative approach that considers the child as a whole, that is, as a mind-body unit. According to this perspective, all the functions are closely interconnected and are organized to cooperate with one another in order to achieve a specific goal, aiming at the optimal adaptation of the child to their surrounding environment. When cooperation between multiple functions is not possible or is difficult, and optimal adaptation of the child to the living environment cannot be achieved, the characteristics of the environment must be adapted to the child's needs and requirements through perceptual-motor facilitation interventions.
- The proposals include an initial phase of observation of the child's attempts to actively experiment with autonomous action strategies. From observing the difficulties the child faces during these attempts, a "facilitating" phase follows, consisting of perceptual-motor guidance to action, which should enable the child to succeed in the actions outlined by the specific proposal. Once the child has mastered the specific skill through facilitating intervention, they are allowed to actively and autonomously experiment with the specific actions prescribed in the proposal, so that, through trial and error, they can select the most effective strategies to achieve their goal. Only after this can the proposal be gradually modified in a "challenging" direction, by progressively adding levels of complexity and increasing difficulty. The increase in the level of challenge can be achieved by modifying the demands, reducing the facilitations used, and requiring the simultaneous control of multiple functions during the same task.
- The proposals that prove effective in producing adaptive changes in the child during therapy should be shared with the family, collaborating to identify strategies for transferring them to the home environment. Family members should be supported in understanding the objectives of the various proposals, paying attention to the child's reactions, and managing the timing of the proposals (e.g., when during the day, in which daily life situation, for how long, how many times per day, etc.).
- The selection of objects and activities, and the adaptation of the context (among the options outlined in the table), are variables that depend on the child's functional level, as indicated by classification scales (VFCS; GMFCS, Mini-MACS). The choice of whether the proposal should be facilitative or challenging, as well as its duration, frequency, and the time to be dedicated to each individual proposal within the rehabilitative plan, must necessarily vary from child to child and, for the same child, even from session to session, based on their interest, needs, motivation levels, and availability. This is done to support the child's motivation and the pleasure of learning.
- The objectives of each activity have been divided and differentiated based on the functional level, according to the classifications indicated by the VFCS scales. For certain skills at level V, it was not possible to identify a reachable objective, and an alternative goal was proposed for that skill.

- In the presence of difficulties with visual tracking by the child, it is recommended to evaluate the use of a checkerboard and/or high-contrast black-and-white images and objects, which can amplify visual perception cues related to the objects in use and the child’s action context. These precautions facilitate the child’s attentional orientation, making it possible to integrate information from the visual channel with the other functions. The checkerboard can be used alone as an attentional cue or as a background for objects to enhance the perception of the objects in relation to the background. Another useful measure for VFCS levels III-IV is the placement of soft lighting in the room (free from direct and intense light sources) and the use of a flashlight to illuminate the child’s or caregiver’s face, or the objects being presented. For VFCS level IV, it is helpful to combine these measures with the use of multisensory objects.
- If a decline in attention and availability is observed, it is useful to introduce novel elements to regain their attention. This can be achieved by alternating the use of objects (from those described) or using them in combination (e.g., face + flashlight; rattle + checkerboard + flashlight, and so on).
- It is advisable to include breaks and activity changes when the child no longer shows interest in the current activity.
- The overall duration of the specific activity proposed is related to the child's achievement of the objective and their motivation to persist in completing it.
- The choice of position the child may maintain during each specific proposal is based on their motor skills and should always allow them to perform at their best, particularly when multiple functions are involved simultaneously. The reference criterion for determining the appropriate level of challenge to present to the child during activities is the "optimal challenge," which provides a reasonable expectation of success. During proposals related to visual-visuomotor functions, it is important to proceed in a facilitative manner with respect to postural control requirements, allowing the child to better direct their resources towards visual tasks.
- The age-based division is indicative, and it is possible to introduce activities and objects from previous age ranges within each specific age group.

Intervention objectives for visual and oculomotor functions

- Eye contact
- Oculomotor functions (fixation, smooth pursuit, saccades)
- Visual attention and interaction
- Perception of near and far space
- Visual localization of people and objects in different spatial areas

Age-appropriate tools

**0-6 months**	**6-12 months**	**12-24 months**	**Contextual elements**

Checkerboard	Castle with battlements or ship with portholes in rigid cardboard (approximately 40x70 cm in size)	Increasing complexity interlocks	Carpet
Flashlight	Spoked wheel	Building blocks	Lighting
Human face	Rotating plate	Everyday objects (plate, spoon, fork, glass, small pot, brush)	Affectionately significant family member
Fantz face	Cubes (1 cm, 2 cm)	Books with windows	Soft containment rolls
Bull's eye	Containers	Animals	Wedge
Necklaces	Rattles	Toy cars	Roller
Graspable ball	Board books	Toy cars with pull-back mechanism	Soft ladder
Softball	Button games	First steps trolley	Cube or table (h 40 cm)
Sensory ball	Top	Sheets/markers	Checkerboard
Bells<br>Ribbons with bells	Musical instruments (keyboard, drum, maracas, rattles, rain stick)	Velcro fruit	Rocking board
Spring	Graspable objects and toys	Rings	Sensory surfaces
Koosh ball	Bimanual articulated objects	Pull-toy with string	Black/white striped surface
Sound ring	Multimodal toys	Images and photos of everyday objects	
Easter egg paper	Napkin to hide objects	Small roller (15 cm diameter)	Music, songs, rhymes

Visual functions chart

**0-3 months**

Ability	VFCS	Objective	Context	Child	Tools	Proposals
**Fixation**	I-II	Increase in the duration of fixation on the target.	Calm environment, with adapted lighting (diffused light, free from direct light sources such as window, chandelier, lamp, etc.)..	1. In the parent's arms, contained position with the practitioner in front.<br>2. Supine, inclined about 30° with the head supported by the adult's hand (open palm), contained distally (feet resting against the adult's abdomen); positions involving flexion and containment are always preferable.<br>3. Prone, with a roll under the chest and the upper limbs free in front.	Target by age group based on the child's interest.	1) Bring your face about 10-20 cm from the child's face, speaking or singing songs while maintaining eye contact for as long as possible; promoting eye contact between the caregiver and the child.<br>2) Present one object at a time, at close distance, seeking the child's gaze starting from their preferred position.<br><br>Facilitation: Guide multimodal integration ("see-listen-touch") through contact with the face or object.
	III-IV	1. Promotion of awareness of residual vision (IV).<br>2. Promotion of fixation (III-IV).<br>3. Increase in the duration of fixation on the target (III).	Calm environment, with adapted lighting (slightly dimmed setting).		High-contrast and/or illuminated (III-IV) or multisensory targets (IV).	
	V	Not evocable fixation:<br>1. Promotion of awareness of residual vision, if present.<br>2. Integrated use of other sensory channels.	Calm environment, with adapted lighting (dimmed).		Multisensory and illuminated targets.	
**Accommodation Convergence**	I-II	1. Maintenance of fixation on the target during approach and withdrawal.<br>2. Increase in fixation on the target during approach and withdrawal.	Calm environment, with adapted lighting (diffused light, free from direct light sources such as window, chandelier, lamp, etc.).	1. In the parent's arms, contained position with the practitioner in front.<br>2. Supine, inclined about 30° with the head supported by the adult's hand (open palm), contained distally (feet resting against the	Target by age group based on the child's interest.	Bring the target in front of the child's face and move it slowly, bringing it closer and further away along the median line (maximum distance of 30 cm) within the child's visual field, until gaze convergence is achieved on the presented

	III-IV	1. Maintenance of fixation on the target during approach and withdrawal.<br>2. Increase in fixation on the target during approach and withdrawal.	Calm environment, with adapted lighting (slightly dimmed setting).	adult's abdomen); positions involving flexion and containment are always preferable.<br>3. Prone, with a roll under the chest and the upper limbs free in front.	High-contrast and/or illuminated (III-IV) or multisensory targets (IV).	object.<br><br>Facilitation: Guide multimodal integration 'see-hear-touch' through contact with the face or object
	V	Not achievable.	-	-	-	-
**Smooth pursuit (horizontal)**	I-II	Horizontal target tracking:<br>1. Tracking from the midline toward one side and back.<br>2. Tracking fully across the horizontal plane.<br>3. Tracking smoothly and completely across the horizontal plane.	Calm environment, with adapted lighting (diffused light, free from direct light sources such as window, chandelier, lamp, etc.).	1. In the parent's arms, contained position with the practitioner in front.<br>2. Supine, inclined about 30° with the head supported by the adult's hand (open palm), contained distally (feet resting against the adult's abdomen); positions involving flexion and containment are always preferable.<br>3. Prone, with a roll under the chest and the upper limbs free in front.<br><br>Targets can be presented starting from the child's preferred head position or by positioning the child with the head and torso aligned.	Target by age group based on the child's interest.	1. Present your face in the primary gaze position (on the midline) at a distance of approximately 10–20 cm from the child's face, seeking their visual engagement. Slowly move to the right/left, ensuring the child maintains visual engagement with your face, and then return to the midline.<br>2. Present an object in the primary gaze position (on the midline) at a distance of approximately 10–20 cm from the child's face, seeking their visual engagement. Slowly move to the right/left, ensuring the child maintains visual engagement with the object, and then return to the midline.<br>3. Present a target in the primary gaze position (on the midline) at a distance of approximately 10–20 cm from
	III-IV	Horizontal target tracking:<br>1. Promotion of target localization.<br>2. Tracking on the horizontal plane.	Calm environment, with adapted lighting (slightly dimmed setting).		High-contrast and/or illuminated (III-IV) or multisensory targets (IV).	
	V	Fixation not achievable:<br>- Promotion of target localization.	Calm environment, with adapted lighting (dimmed).		Multisensory and illuminated targets.	

						the child's face. Slowly move the target along the entire horizontal arc.<br><br>Facilitation: Guide multimodal integration ("see-hear-touch") through contact with the face or object (e.g., speaking or singing nursery rhymes).
**Smooth pursuit (vertical)**	I-II	Vertical target tracking:<br>1. Tracking the target from the midline upward/downward and back.<br>2. Tracking fully across the vertical plane.<br>3. Tracking smoothly and completely across the vertical plane.	Calm environment, with adapted lighting (diffused light, free from direct light sources such as window, chandelier, lamp, etc.).	1. In the parent's arms, contained position with the practitioner in front.<br>2. Supine, inclined about 30° with the head supported by the adult's hand (open palm), contained distally (feet resting against the adult's abdomen); positions involving flexion and containment are always preferable.<br><br>Targets can be presented starting from the child's preferred head position or by positioning the child with the head and torso aligned.	Target by age group based on the child's interest.	1. Present your face in the primary gaze position (on the midline) at a distance of approximately 10–20 cm from the child's face, seeking their visual engagement. Slowly move upward/downward, ensuring the child maintains visual engagement with your face, and then return to the midline.<br>2. Present an object in the primary gaze position (on the midline) at a distance of approximately 10–20 cm from the child's face, seeking their visual engagement. Slowly move upward/downward, ensuring the child maintains visual engagement with the object, and then return to the midline.<br>3. Present a target in the primary gaze position (on the midline) at a distance of approximately 10–20 cm from
	III-IV	Vertical target tracking:<br>1. Promotion of target localization.<br>2. Tracking on the vertical plane.	Calm environment, with adapted lighting (slightly dimmed setting).		High-contrast and/or illuminated (III-IV) or multisensory targets (IV).	
	V	Fixation not achievable:<br>- Promotion of target localization.	Calm environment, with adapted lighting (dimmed).		Multisensory and illuminated targets.	

						the child's face. Slowly move the target along the entire vertical arc.<br><br>Facilitation: Guide multimodal integration ("see-hear-touch") through contact with the face or object (e.g., speaking or singing nursery rhymes).
**3-6 months**						
**Ability**	**VFCS**	**Objective**	**Context**	**Child**	**Tools**	**Proposals**
**Attraction saccade (center-periphery game)**	I-II	Promotion of target localization through attraction saccades:<br>1. Reduction in starter latency.<br>2. Improvement in the accuracy of saccadic movements.	Calm environment, with adapted lighting (diffused light, free from direct light sources such as window, chandelier, lamp, etc.).	1. In the parent's arms, contained position with the practitioner in front.<br>2. Supine, inclined about 30° with the head supported by the adult's hand (open palm), contained distally (feet resting against the adult's abdomen); positions involving flexion and containment are always preferable.<br><br>Targets can be presented starting from the child's preferred head position or by positioning the child with the head and torso aligned.	Target by age group based on the child's interest.	1. Present two games in the primary gaze position, one hidden behind the other, engaging the child's fixation on the visible target. Move the hidden target away from the other by at least 5 cm (in various directions in space), making it suddenly appear within the child's view, encouraging the child to shift fixation from the first to the second object.<br>2. Present the target in the primary gaze position at a distance of about 20-30 cm, engaging the child's fixation on it. Make the second target appear from the periphery of the visual field (alternating from right/left/top/bottom), moving it
	III-IV	Promotion of target localization through attraction saccades:<br>1. Reduction in starter latency.<br>2. Improvement in the accuracy of saccadic movements.	Calm environment, with adapted lighting (slightly dimmed setting).		High-contrast and/or illuminated (III-IV) or multisensory targets (IV).	

						slowly along the arc of the visual field towards the primary gaze position, until the child notices it and shifts their gaze towards it.
	V	Not achievable.	-	-	-	-
**Fixation**	I-II	Increase in the duration of fixation on the target.	Calm environment, with adapted lighting (diffused light, free from direct light sources such as window, chandelier, lamp, etc.).	1. In the parent's arms, contained position with the practitioner in front.<br>2. Supine, inclined about 30° with the head supported by the adult's hand (open palm), contained distally (feet resting against the adult's abdomen); positions involving flexion and containment are always preferable.<br>3. Prone.<br>4. Seated position on the floor with containment.	Target by age group based on the child's interest.	1. Bring your face to a distance of about 30 cm from the child's face, speaking or singing nursery rhymes, while maintaining the fixation for as long as possible; promote eye contact between the caregiver and the child.<br>2. Present one object at a time, at a close distance, seeking the child's gaze starting from their preferred position.<br><br>Facilitation: Guide multimodal integration ("see-listen-touch") through contact with the face or object.
	III-IV	1. Promotion of awareness of residual vision (IV).<br>2. Promotion of fixation (III-IV).<br>3. Increase in the duration of fixation on the target (III).	Calm environment, with adapted lighting (slightly dimmed setting).		High-contrast and/or illuminated (III-IV) or multisensory targets (IV).	
	V	Non-evocable fixation:<br>1. Promotion of awareness of residual vision, if present.<br>2. Integrated use of other sensory channels.	Calm environment, with adapted lighting (dimmed).		Multisensory and illuminated targets.	
**Smooth pursuit (horizontal)**	I-II	Horizontal target tracking:<br>1. Tracking from the	Calm environment, with adapted lighting (diffused light, free	1. In the parent's arms, contained position with the practitioner in front	Target by age group based on the child's interest.	1. Present your face in the primary gaze position (on the midline) at a distance of

		midline toward one side and back.<br>2. Tracking fully across the horizontal plane.<br>3. Tracking smoothly and completely across the horizontal plane.	from direct light sources such as window, chandelier, lamp, etc.).	2. Supine, inclined about 30° with the head supported by the adult's hand (open palm), contained distally (feet resting against the adult's abdomen); positions involving flexion and containment are always preferable.<br>3. Prone.<br>4. Seated position on the floor with containment.<br><br>Targets can be presented starting from the child's preferred head position or by positioning the child with the head and torso aligned.		approximately 30 cm from the child's face, seeking their visual engagement. Slowly move to the right/left, ensuring the child maintains visual engagement with your face, and then return to the midline.<br>2. Present an object in the primary gaze position (on the midline) at a distance of approximately 30 cm from the child's face, seeking their visual engagement. Slowly move to the right/left, ensuring the child maintains visual engagement with the object, and then return to the midline.<br>3. Present a target in the primary gaze position (on the midline) at a distance of approximately 30 cm from the child's face. Slowly move the target along the entire horizontal arc.<br><br>Facilitation: Guide multimodal integration ("see-hear-touch") through contact with the face or object (e.g., speaking or singing nursery rhymes).
	III-IV	Horizontal target tracking:<br>1. Promotion of target localization.<br>2. Tracking on the horizontal plane.	Calm environment, with adapted lighting (slightly dimmed setting).		High-contrast and/or illuminated (III-IV) or multisensory targets (IV).	
	V	Fixation not achievable:<br>- Promotion of target localization.	Calm environment, with adapted lighting (dimmed).		Multisensory and illuminated targets.	
**Smooth pursuit (vertical)**	I-II	Vertical target tracking:<br>1. Tracking the target from the midline upward/downward and back.	Calm environment, with adapted lighting (diffused light, free from direct light sources such as	1. In the parent's arms, contained position with the practitioner in front<br>2. Supine, inclined about 30° with the head supported by	Target by age group based on the child's interest.	1. Present your face in the primary gaze position (on the midline) at a distance of approximately 30 cm from the child's face, seeking their visual

		2. Tracking fully across the vertical plane.<br>3. Tracking smoothly and completely across the vertical plane.	window, chandelier, lamp, etc.).	the adult's hand (open palm), contained distally (feet resting against the adult's abdomen); positions involving flexion and containment are always preferable.<br>3. Prone.<br>4. Seated position on the floor with containment.<br><br>Targets can be presented starting from the child's preferred head position or by positioning the child with the head and torso aligned.		engagement. Slowly move upward/downward, ensuring the child maintains visual engagement with your face, and then return to the midline.<br>2. Present an object in the primary gaze position (on the midline) at a distance of approximately 30 cm from the child's face, seeking their visual engagement. Slowly move upward/downward, ensuring the child maintains visual engagement with the object, and then return to the midline.<br>3. Present a target in the primary gaze position (on the midline) at a distance of approximately 30 cm from the child's face. Slowly move the target along the entire vertical arc.<br><br>Facilitation: Guide multimodal integration ("see-hear-touch") through contact with the face or object (e.g., speaking or singing nursery rhymes).
	III-IV	Vertical target tracking:<br>1. Promotion of target localization.<br>2. Tracking on the vertical plane.	Calm environment, with adapted lighting (slightly dimmed setting).		High-contrast and/or illuminated (III-IV) or multisensory targets (IV).	
	V	Fixation not achievable:<br>- Promotion of target localization.	Calm environment, with adapted lighting (dimmed).		Multisensory and illuminated targets.	
**Oculomotor coordination**	Please refer to the praxic function.					
**6-12 months**						
**Ability**	**VFCS**	**Objective**	**Context**	**Child**	**Tools**	**Proposals**

**Saccadic movements**	I-II	Promotion of target localization through saccades:<br>1. Reduction in starter latency.<br>2. Improvement in the accuracy of saccadic movements.	Calm environment, with adapted lighting (diffused light, free from direct light sources such as window, chandelier, lamp, etc.).	1. In the parent's arms, contained position with the practitioner in front<br>2. Supine, inclined about 30° with the head supported by the adult's hand (open palm), contained distally (feet resting against the adult's abdomen); positions involving flexion and containment are always preferable.<br>3. Seated position on the floor with containment.<br>4. Seated on the floor or on a small bench.	Castle/Ship + Target by age group based on the child's interest.	1. Use the Castle/Ship to make characters appear through different holes, encouraging the child to perform saccadic movements to locate them. It is also possible to allow the child to grasp the objects.<br>Other similar activities can be created and proposed at the discretion of the caregiver, as long as they are embedded in a playful, narrative, and motivating context.<br>Facilitation: Choose an object with sufficient contrast to the Castle/Ship, and if necessary, illuminate it or use luminous objects.
	III-IV	Promotion of target localization through saccades:<br>1. Reduction in starter latency.<br>2. Improvement in the accuracy of saccadic movements.	Calm environment, with adapted lighting (slightly dimmed setting).		Castle/Ship + High-contrast and/or illuminated (III-IV) or multisensory targets (IV).	
	V	Not achievable.	-	-	-	-
**Oculomotor coordination**	Please refer to the praxic function.					
**12-18 months**						
**Ability**	**VFCS**	**Objective**	**Context**	**Child**	**Tools**	**Proposals**
**Smooth pursuit (variable direction)**	I-II	Target tracking in all directions:<br>1. Tracking fully in all directions of gaze.<br>2. Tracking smoothly and completely in all	Calm environment, with adapted lighting (diffused light, free from direct light sources such as window, chandelier,	Identify a position that the child can maintain stably and/or that does not require excessive postural control, so they can focus on utilizing the visual channel.	Target by age group based on the child's interest.	Propose activities for the child to track an object moving slowly through space. Possible examples include: a car/ball rolling along an inclined plane; a small boat moving in water; a

		directions of gaze.	lamp, etc.).	1. Seated in a postural support system.<br>2. Seated on the floor with support.<br>3. Seated on the floor or on a small bench.		train running along a track, etc.<br><br>Other similar activities can be created and proposed as long as they are embedded in a playful, narrative, and motivating context.
	III-IV	Target tracking in all directions:<br>1. Promotion of target localization.<br>2. Tracking in all directions of gaze.	Calm environment, with adapted lighting (slightly dimmed setting).		High-contrast and/or illuminated (III-IV) or multisensory targets (IV).	
	V	Not achievable.	-	-	-	-
**Saccadic exploration**	I-II	Use of saccadic eye movements for spontaneous exploration in the peripersonal and extrapersonal space.	Calm environment, with adapted lighting (diffused light, free from direct light sources such as window, chandelier, lamp, etc.).	Identify a position that the child can maintain stably and/or that does not require excessive postural control, so they can focus on utilizing the visual channel.<br><br>1. Seated in a postural support system.<br>2. Seated on the floor with support.<br>3. Seated on the floor or on a small bench.	Target by age group based on the child's interest.	1. Visual exploration of an image (e.g., flipping through a book).<br>2. Guided exploration of items placed on a surface (e.g., table) in peripersonal space (e.g., searching for blocks to build a tower, finding a shape to fit in the correct slot).<br>3. Visual exploration of the environment – searching for objects upon request (e.g., "Throw the ball to…") in various directions to at least 2/3 people.<br>Facilitations/strategies: Arrange elements aligned with each other and then distribute them randomly on the surface, gradually increasing the number of items presented, start with simple images with few details and progressively introduce images with more
	III-IV	Use of saccadic eye movements for spontaneous exploration in the peripersonal space.	Calm environment, with adapted lighting (slightly dimmed setting).		High-contrast and/or illuminated (III-IV) or multisensory targets (IV).	

						visual complexity, use pointing cues.
	V	Not achievable.	-	-	-	-
**18-24 months**						
**Ability**	**VFCS**	**Objective**	**Context**	**Child**	**Tools**	**Proposals**
**Smooth pursuit (variable direction)**	I-II	Target tracking in all directions:<br>1. Tracking fully in all directions of gaze.<br>2. Tracking smoothly and completely in all directions of gaze.	Calm environment, with adapted lighting (diffused light, free from direct light sources such as window, chandelier, lamp, etc.).	Identify a position that the child can maintain stably and/or that does not require excessive postural control, so they can focus on utilizing the visual channel.<br><br>1. Seated in a postural support system.<br>2. Seated on the floor with support.<br>3. Seated on the floor or on a small bench.	Target by age group based on the child's interest.	Propose activities for the child to track an object moving slowly through space. Possible examples include: a car/ball rolling along an inclined plane; a small boat moving in water; a train running along a track, etc.<br><br>Other similar activities can be created and proposed as long as they are embedded in a playful, narrative, and motivating context.
	III-IV	Target tracking in all directions:<br>1. Promotion of target localization.<br>2. Tracking in all directions of gaze.	Calm environment, with adapted lighting (slightly dimmed setting).		High-contrast and/or illuminated (III-IV) or multisensory targets (IV).	
	V	Not achievable.	-	-	-	-
**Saccadic exploration**	I-II	Use of saccadic eye movements for spontaneous exploration in the peripersonal and extrapersonal space.	Calm environment, with adapted lighting (diffused light, free from direct light sources such as window, chandelier, lamp, etc.).	Identify a position that the child can maintain stably and/or that does not require excessive postural control, so they can focus on utilizing the visual channel.	Target by age group based on the child's interest.	1. Visual exploration of an image (e.g., flipping through a book).<br>2. Guided exploration of items placed on a surface (e.g., table) in peripersonal space (e.g., searching for blocks to build a

	III-IV	Use of saccadic eye movements for spontaneous exploration in the peripersonal space.	Calm environment, with adapted lighting (slightly dimmed setting).	1. Seated in a postural support system.<br>2. Seated on the floor with support.<br>3. Seated on the floor or on a small bench.	High-contrast and/or illuminated (III-IV) or multisensory targets (IV).	tower, finding a shape to fit in the correct slot).<br>3. Visual exploration of the environment – searching for objects upon request (e.g., “Throw the ball to…”) in various directions to at least 2/3 people.<br><br>Facilitations/strategies: Arrange elements aligned with each other and then distribute them randomly on the surface, gradually increasing the number of items presented, start with simple images with few details and progressively introduce images with more visual complexity, use pointing cues.
	V	Not achievable.	-	-	-	-
**Oculomotor coordination**	Please refer to the praxic function.					
