# Supplementary material for: A Multidimensional and Integrated Rehabilitation Approach (A.M.I.R.A.) for Infants at Risk of Cerebral Palsy and Other Neurodevelopmental Disabilities
Source: Children (Basel). 2025 Jul 30;12(8):1003. doi: 10.3390/children12081003 (PMC12384761; doi:10.3390/children12081003)
Supplement: Supplementary file 1 [file children-12-01003-s001.zip › Table S4 - Postural-Motor Function Chart.pdf]

Table S4 - Postural-Motor Function Chart

Premises for using the chart

- All the proposals described below refer to a rehabilitative approach that considers the child as a whole, that is, as a mind-body unit. According to this perspective, all the functions are closely interconnected and are organized to cooperate with each other in order to achieve a specific goal, aiming at the optimal adaptation of the child to their surrounding environment. When cooperation between multiple functions is not possible or is difficult, and the optimal adaptation of the child to the living environment cannot be achieved, the characteristics of the environment must be adapted to the child's needs and requirements through perceptual-motor facilitation interventions.
- The proposals include an initial phase of observation of the child’s attempts to actively experiment with autonomous action strategies. From observing the difficulties the child faces during these attempts, a "facilitating" phase follows, consisting of perceptual-motor guidance to action, which should enable the child to succeed in the actions outlined by the specific proposal. Once the child has mastered the specific skill through facilitating intervention, they are allowed to actively and autonomously experiment with the specific actions prescribed in the proposal, so that, through trial and error, they can select the most effective strategies to achieve their goal. Only after this can the proposal be gradually modified in a "challenging" direction, by progressively adding levels of complexity and increasing difficulty. The increase in the level of challenge can be achieved by modifying the demands, reducing the facilitations used, and requiring the simultaneous control of multiple functions during the same task.
- The proposals that prove effective in producing adaptive changes in the child during therapy should be shared with the family, collaborating to identify strategies for transferring them to the home environment. Family members should be supported in understanding the objectives of the various proposals, paying attention to the child’s reactions, and managing the timing of the proposals (e.g., when during the day, in which daily life situation, for how long, how many times per day, etc.).
- The selection of objects and activities, and the adaptation of the context (from the options outlined in the table), are variables that depend on the child's functional level, as indicated by classification scales (VFCS; GMFCS, Mini-MACS). The choice of whether the proposal should be facilitative or challenging, as well as its duration, frequency, and the time to be dedicated to each individual proposal within the rehabilitative plan, must necessarily vary from child to child and, for the same child, even from session to session, based on their interest, needs, motivation levels, and availability. This is done to support the child's motivation and the pleasure of learning.
- In the presence of difficulties with visual tracking by the child, it is recommended to evaluate the use of a checkerboard and/or high-contrast black-and-white images and objects that can amplify the visual perception cues related to the objects in use and the child’s action context. These precautions help facilitate the child's attentional orientation, making it possible to integrate information from the visual channel with the other functions. The checkerboard can be used alone as an attentional cue or

as a background for objects to enhance the perception between objects and the background. Another useful measure is the placement of soft lighting in the room (free from direct and intense light sources) and the use of a flashlight to illuminate the child's or caregiver's face, or the objects being presented.

- If a decline in attention and availability is observed, it is useful to introduce novel elements to regain their attention. This can be achieved by alternating the use of objects (from those described) or using them in combination (e.g., face + flashlight; rattle + checkerboard + flashlight, and so on).
- It is advisable to include breaks and activity changes when the child no longer shows interest in the current activity.
- The overall duration of the specific activity proposed is related to the child's achievement of the objective and their motivation to persist in completing it.
- The choice of position the child may maintain during each specific proposal is based on their motor skills and should always allow them to perform at their best, particularly when multiple functions are involved simultaneously. The reference criterion for determining the appropriate level of challenge to present to the child during activities is the "optimal challenge," which provides a reasonable expectation of success.
- In the specific intervention on postural-motor function, the approach always starts in a facilitative manner until a given skill is acquired, after which it gradually progresses in a challenging direction once the control of that specific ability has been achieved and stabilized. The postures indicated in the tables show a progression from facilitative conditions to challenging conditions.
- The use of braces and assistive devices should be evaluated on a case-by-case basis and introduced, if necessary, as an integral part of the rehabilitative plan as a facilitative proposal.
- The age-based division is indicative, and it is possible to introduce activities and objects from previous age ranges within each specific age group.

Objectives for Postural-Motor Function

- Ability to control different postures in static and dynamic situations (during perturbations either induced by the therapist or self-induced by the child in an attempt to establish eye contact and/or reach objects of interest) and to vary posture when transitioning between different positions (supine, prone, seated, etc.);
- Ability to vary and control movements of the head, trunk, lower limbs, and upper limbs during various activities;
- Ability to move autonomously on the ground: prone or seated, with or without simple assistive devices (e.g., baby walker, pedal-less tricycle, triangle or similar devices, etc.);
- Ability to move from a standing position with support, with or without assistive devices, etc.;
- Ability to ascend and descend stairs, overcome small obstacles, etc.;
- Autonomous locomotion with changes in direction and dragging or pushing objects (e.g., cart, toys) with or without assistive devices or orthoses, etc.

Age-appropriate tool

0-6 months	6-12 months	12-24 months	Contextual elements
Checkerboard	Multimodal toys	Puzzles with increasing complexity	Mat
Flashlight	Radiating structure	Building blocks	Lighting
Human face	Rotating plate	Everyday objects (plate, spoon, fork, cup, small pot, brush)	Emotionally significant familiar item
Fantz face	Cubes (1 cm, 2 cm)	Books with flaps	Soft containment roller
Bull's eye	Containers	Animals	Wedge
Necklaces	Rattles	Toy cars	Roller
Graspable ball	Soft books	Wind-up toy cars	Soft ladder
Soft ball	Button-activated toys	First-step stroller	Cube or table (40 cm height)
Sensory ball	Spinning top	Sheets/markers	Checkerboard
Jingle bells	Musical instruments (keyboard, drum, maracas, rattles, rainstick)	Velcro fruits	Rocking board
Spring	Graspable objects and toys	Rings	Sensory surfaces
Koosh ball	Bimanually articulated objects	Pull toys with strings	Black/white striped surface
Sound ring	Multimodal toys	Images and photos of everyday objects	High-contrast color spokes
Ribbons with bells	Napkin for hiding objects	Small roller (15 cm in diameter)	Music, songs, nursery rhymes
Aluminum foil	Cause-and-effect toys	Containers of various sizes	Handrails and support bars

Postural-Motor Function chart

0-6 months						
**Ability**	**GMFCS**	**Objective**	**Context**	**Child**	**Tools**	**Proposals**
**Organization along the midline** (2-3 months)	NA	In a supine position on a mat, the child maintains head and trunk alignment and orients the limbs toward the midline.	Quiet environment, with adapted lighting: dimmed or slightly dimmed or diffused light (free from direct light sources such as window, chandelier, lamp, etc.), free from distracting or confusing factors.<br>Use of a baby bouncer, wedge, or containment rolls.	1. Supine, contained (in bouncer, wedge, or nest).<br>2. Supine on mat, free.	Soft, bright, and/or auditory or high-contrast objects.	In the selected position, actively engage the child's attention and maintain eye contact from a close distance, using voice if necessary, to promote postural alignment through visual tracking.<br>Present an object along the midline and within the child's visual field, in contact with the abdomen and hands; if needed, guide the hands to reach and explore the object.<br>In the selected position, present an object along the midline and within the child's visual field, waiting for the child to orient the hands toward the object until contact is made.
**Prone, lifting the head** (2 months)	NA	From the prone position, the child lifts their head to establish and maintain eye contact with the caregiver or an object of interest.	Quiet environment, with adapted lighting: dimmed or slightly dimmed or diffused light (free from direct light sources such as window, chandelier, lamp, etc.), free from distracting or confusing factors.<br><br>Use of wedge and/or checkerboard as a vertical surface on the	1. Prone on the wedge (parallel to the incline, with the head positioned at the upper part).<br>2. Prone on the wedge with a roll under the chest and armpits to assist in maintaining the upper limbs in the anterior space for support.<br>3. Prone on a mat.	Age-appropriate objects.	In the selected position, engage the child's attention and maintain eye contact in the anterior space, also providing verbal support to assist in the anti-gravity lifting of the head.

			frontal plane.			
**Prone, lifting the head and chest while extending the elbows** (3 months)	NA	From the prone position, the child lifts their head and chest by extending the arms to establish and maintain eye contact with the caregiver or an object of interest.	Quiet environment, with adapted lighting: dimmed or slightly dimmed or diffused light (free from direct light sources such as window, chandelier, lamp, etc.), free from distracting or confusing factors.<br><br>Use of wedge and/or checkerboard as a vertical surface on the frontal plane.	1. Prone on the wedge (parallel to the incline, with the head positioned at the upper part).<br>2. Prone on the wedge with a roll under the chest and armpits to assist in maintaining the upper limbs in the anterior space for support.<br>3. Prone on a mat.	Age-appropriate objects.	In the selected position, engage the child's attention and maintain eye contact in the anterior space, providing verbal support to assist in the anti-gravity lifting of the head and in maintaining the position.
**Half roll** (3 months)	NA	From the supine position, the child autonomously reaches a side-lying position (right or left).	Quiet environment, with adapted lighting: dimmed or slightly dimmed or diffused light (free from direct light sources such as window, chandelier, lamp, etc.), free from distracting or confusing factors.<br><br>Use of wedge and/or checkerboard as a landing surface.	1. Supine on the wedge, perpendicular to the incline (the presence of the slope amplifies the outcomes of the child's spontaneous initiative and rolling attempts, enhancing their sense of self-efficacy).<br>2. Supine on a mat, free.	Age-appropriate objects.	Propose the activities after positioning the child on the wedge, actively engaging and maintaining eye contact with the child at close range, using voice in a pleasant interaction climate. Slowly move laterally, encouraging the child to follow with their gaze, and wait for the child's initiative to either maintain or re-establish visual-tactile contact with the adult or the motivational object placed to the side.<br>Assist the child, if needed, in maintaining the side-lying position.<br>If necessary, manually guide the child through the rolling movement.<br>Start the activity from the supine position on the mat.

**Rolling**<br>(4-5 months as a block, from 6 months as a fluid sequence with trunk rotation)	NA	From the supine position, the child rolls to the prone position to achieve a goal.	Quiet environment, with adapted lighting: dimmed or slightly dimmed or diffused light (free from direct light sources such as window, chandelier, lamp, etc.), free from distracting or confusing factors.<br><br>Use of wedge and/or checkerboard as a landing surface.	1. Supine on the wedge, perpendicular to the incline (the presence of the slope amplifies the outcomes of the child's spontaneous initiative and rolling attempts, enhancing their sense of self-efficacy).<br>2. Supine on a mat, free.	Age-appropriate objects.	See the "Half roll" function.<br>Once the child has reached the side-lying position, move or place the object of interest on the plane in the antero-superior space so that the child is encouraged to complete the roll to the prone position in order to maintain or re-establish visual-tactile contact with the object of interest.<br>Assist with manual guidance in performing the rolling movement, if needed.<br>Once the roll is complete, help the child free the upper limb, if necessary.
**Pivoting from prone**<br>(6 months)	NA	In the prone position, the child can rotate on their belly, pivoting on their limbs.	Quiet environment, with adapted lighting: dimmed or slightly dimmed or diffused light (free from direct light sources such as window, chandelier, lamp, etc.), free from distracting or confusing factors.<br><br>A turntable positioned above a radial platform.	1. Prone, lying with the abdomen on the turntable to eliminate friction between the body and the surface, facilitating pivoting movement.<br>2. Prone on the mat.	Radial surface, turntable, mat.	On the mat, place a multimodal object that piques the child's interest on the high-contrast radial surface at a 45° angle to the side of the child (first on the right, then on the left) so that it can be reached by activating pivoting from the prone position, aided by the reduced friction with the floor due to the turntable beneath.<br>Make the same proposal on the mat without the turntable.
**Creeping**<br>(6 months)	I-II	From the prone position, the child	Quiet environment, with adapted lighting:	Prone.	Black/white striped surface to facilitate	Present an object or the face in the anterior space, using the chessboard as a

	III-IV	progresses forward to reach an object of interest placed 10-15 cm away.	dimmed or slightly dimmed or diffused light (free from direct light sources such as window, chandelier, lamp, etc.), free from distracting or confusing factors.<br><br>Black/white striped surface applied to a horizontal plane or on the wedge (stripes parallel to the direction of the incline).	Prone on the wedge placed in favor of gravity (downward) to facilitate performance.	visual perception of movement progression in space.<br><br>Age-appropriate objects.	background if necessary, while verbally supporting the child in reaching it. Wait for and encourage the child's spontaneous, independent attempts, allowing them to actively experiment with their own movement strategies. Offer your hand against the soles of their feet to facilitate propulsion. If helpful, place the child on the wedge in the indicated position to facilitate sliding toward the object and encourage them to reach the target of interest placed in the anterior space. Position the high-contrast striped surface under the child's abdomen to help with the perception of progression in the anterior space.
	V			Not achievable independently; the movement can be experienced in a playful manner using a cloth.		
**6-10 months**						
**Ability**	**GMFCS**	**Objective**	**Context**	**Child**	**Tools**	**Proposals**
**Sitting position control**<br>(8-9 months)	I-II	Maintaining control of the sitting position with and without the support of the upper limbs.	Mat.<br>Rocking board.	Seated	Human face.<br>Checkerboard.<br>Age-appropriate objects.	If the child is sitting on the mat but unable to maintain the seated position independently, place them sitting on the chessboard to facilitate the initiation of support reactions on it and help maintain the position with support.<br>Create small shifts in the support plane in various directions, triggering the child's balance reactions and parachute reflexes as they attempt to maintain control of the sitting position, while singing rhythmic songs or rhymes, or while observing an engaging scene.<br>Only later should the activity be proposed without the support of the upper limbs.
	III-IV			Seated with pelvic support.		
	V			Not achievable on the mat, but requires a postural support system.		

**Pivoting from sitting** (9-10 months)	I-II	In the seated position on the mat, the child reaches for an object of interest placed behind them by rotating their trunk and pivoting on their sitting position.	Quiet environment, with adapted lighting: dimmed or slightly dimmed or diffused light (free from direct light sources such as window, chandelier, lamp, etc.), free from distracting or confusing factors.<br><br>A turntable positioned on the mat, above a black-and-white radial panel.	Seated on the mat.	Black/white radial surface.<br>Turntable. Age-appropriate objects.	Place a multimodal object of interest on the high-contrast radial surface at a 45° angle behind the child (first on the right, then on the left) so that it can be reached by activating pivoting from the seated position, facilitated by the presence of the turntable.<br>Then, place the object in the same position (45° behind the child) without the turntable and radial surface, encouraging the child to reach it by activating pivoting from the seated position on their own.<br>If needed, manually guide the child to push their feet against the floor and rotate their pelvis to assist in the movement.
	III-IV			Seated on the turntable with pelvic support.		
	V			Not achievable.		
**Supine to sitting position** (7-8 months)	I-II	From the supine position, the child reaches the sitting position by rotating onto one side (right or left).	Quiet environment, with adapted lighting: dimmed or slightly dimmed or diffused light (free from direct light sources such as window, chandelier, lamp, etc.), free from distracting or confusing factors.<br><br>Wedge.	Supine.	Age-appropriate objects.<br>Checkerboard to enhance the perception of the support space.<br>Wedge.	Encourage and motivate the child to lift themselves from the supine position to sit and play with the adult or an object of interest. Facilitate the movement, if necessary, by using the wedge (to reduce the range of motion and gravitational impact) and providing manual guidance (stabilizing the pelvis with one hand while guiding the lateral rotation movement with the other).<br>Pay attention to the child's participation in the movement, ensuring they use their upper limb for support on the surface. If helpful, add the checkerboard to enhance the perceptual awareness of the support plane.
	III-IV			III: lateral decubitus.<br>IV: supine on the wedge.		

	V	Not achievable.	-	-	-	-
**Sitting to crawling transition** (8 months)	I-II	From the seated position, the child transitions to crawling by rotating onto one side (right or left), activating support reactions on the upper limbs.	Quiet environment, with adapted lighting: dimmed or slightly dimmed or diffused light (free from direct light sources such as window, chandelier, lamp, etc.), free from distracting or confusing factors.<br><br>Soft ladder. Roller.	Seated on the mat in long sitting.	Age-appropriate objects.<br>Soft ladder. Roller.	Place an object of interest in the extrapersonal space (reachable by shifting the body's center of mass) in the lateral space (right or left) to encourage the child to transition from a symmetrical seated position to an asymmetrical seated position, with the upper limb of the side of movement in support.<br>Place an object of interest on the steps of the soft ladder to encourage the child to move into the quadruped position on an elevated surface (step) while attempting to reach the object.<br>If helpful, place a chessboard under the object to enhance the "figure-ground" perception.<br>Assist the child, if necessary, by manually guiding the rotation of the pelvis.<br>Position a roller under the child's abdomen, if helpful, to facilitate maintaining the quadruped position.
	III-IV			III: seated on the heels.<br>IV: seated on the heels with pelvic support.		
	V	Not achievable.	-	-	-	-
**Seated position control on a**	I-II	Maintain control of the seated position	Quiet environment, free from distracting or	Seated on the roller.	Caregivers.<br>Age-appropriate	Present an object of interest in the extrapersonal space (reachable by shifting

**small roller**<br>(8–9 months)	III-IV	on the roller, even in the presence of self- and externally-induced imbalances.	confusing factors.<br>Roller.	III: seated on the roller, supported at the lower trunk.<br>IV: seated on the semi-sphere with support.	objects.<br>Roller.	the body's center of mass) in the lateral space (right and left), as well as above and below, to stimulate trunk equilibrium reactions, vertical recovery, and weight-bearing balance on the lower limbs (self-induced imbalances).<br>Gently prompt the same reactions by introducing small perturbations to the roller while singing rhythmic songs and nursery rhymes (externally induced imbalances).
	V	Not achievable.	-	-	-	-
**Maintaining the quadruped position**<br>(8 months)	I-II	Maintain the position supported on four points.	Quiet environment, free from distracting or confusing factors.<br>Soft ladder. Roller.	In an autonomous quadruped position.	Age-appropriate objects, preferably for looking at (without characteristics of graspability), just outside the child's peripersonal space.<br>Checkerboard to amplify the perception of "figure-ground."<br>Tactile surfaces.	In the indicated position, facilitate the child in maintaining the position itself by offering an object of motivational interest placed in the anterior space. If necessary, place a checkerboard or a tactile sensory surface under the child's hands to amplify their perception of weight and assist with opening.
	III-IV			III: in quadruped with upper limbs supported on the first step of the ladder.<br>IV: in quadruped with a support roll positioned under the chest.		
	V	Not achievable.	-	-	-	-
**Transition from the seated**	I-II	Reach an object placed on an	Mat.<br>Step.	Seated on the mat.	Age-appropriate objects.	Activate a multimodal toy placed on the first/second step of the ladder,

**position on the floor to kneeling**<br>(9 months)	III-IV	elevated surface		III: provide lateral or anterior support (e.g., step, backrest, chair, small bench).<br>IV: assist the child in the postural transition with manual support from the practitioner.	Step.	encouraging the child to reach it by transitioning from a seated position to a kneeling one with pelvic rotation.
	V	Not achievable.	-	-	-	-
**Transition from the seated position on the mat to the standing position**<br>(9-10 months)	I-II	Reach an object of interest placed on an elevated surface.	Mat.<br>Cube or table with a height of 40 cm.<br>Support bar attached to the edge of the table.	Seated on the mat.	Age-appropriate objects.<br>Cube.<br>Table.<br>Support bar attached to the support surface.	Activate a multimodal toy placed on the surface, encouraging the child to reach it by transitioning from the sitting position to kneeling, then to a mounted position, and finally to standing. Manually assist the child in the movement, if necessary. If needed, apply a support bar parallel to the edge of the table to facilitate the use of the upper limbs for grasping during the positional transition.
	III-IV			III: provide lateral or anterior support (e.g., ladder, backrest, chair, small bench).<br>IV: assist the postural transition with the operator's support.		
	V	Not achievable.	-	-	-	-
**10-24 months**						
**Ability**	**GMFCS**	**Objective**	**Context**	**Child**	**Tools**	**Proposals**
**Maintaining the sitting position on a small bench**<br>(10 - 11 months)	I-II	Use partial load on the lower limbs to maintain balance during grasping tasks while sitting on the bench.	Bench.	Seated on the bench.	Age-appropriate graspable objects.<br>Small raised platform to lift objects slightly off the floor for	Place several motivating objects of interest on the floor in front of the child, encouraging them to reach for them by shifting weight onto the lower limbs. If needed, place the objects on a raised platform and gradually lower them to the
	III-IV			III: lateral support.<br>IV: posterior and/or lateral support.		

					reaching.<br>Sensory tactile mats.	floor. If necessary, use sensory tactile mats under the feet to help with the perception of weight distribution.
	V	Not achievable.	-	-	-	-
**Passage from sitting position on a small bench to standing position**<br>(11 - 12 months)	I-II	From the sitting position on the bench, move to standing with anterior support.	Table (at chest height when standing).<br>Support bar attached to the edge of the table.<br>Bench.	Seated on the bench.	Age-appropriate objects.<br>Table.<br>Support bar.	Interact with the game placed on the table to motivate the child to reach and activate it themselves. If necessary, guide the child's upper limbs towards the support bar placed on the table.<br>If needed, support the pelvis to stabilize and guide the child in the movement of standing up from the bench.<br>If necessary, use sensory tactile mats as a surface of support under the feet to help with the perception of weight distribution.
	III-IV			III: lateral and/or anterior support.<br>IV: lateral and/or anterior support with operator guidance.		
	V	Not achievable.	-	-	-	-
**Maintaining the standing position with support**	I-II	Maintain the standing position with support on a plane placed anteriorly.	Table or bench at the child's chest height.<br>Support bar attached to the edge of the table.	Standing with support.	Age-appropriate objects (non-graspable, only for looking at).	Present an object of motivational interest that is only to be observed, in order to avoid conflict between the need to keep the upper limbs supported on the surface and the desire to grasp the object.<br>If necessary, apply the support bar to the table to make the upper limbs' grip more effective.<br>If necessary, assist the child manually to
	III-IV			III: Standing with support on the anterior plane.<br>IV: Attempt standing with support on the anterior plane with stabilization of the lower limbs.		

	V			Guide the child in using the table for static support.		maintain symmetrical body alignment while supporting the load.
**Maintaining the standing position without support**	I-II	Maintain the standing position, adjusting the weight distribution to auto- or externally induced oscillations.	Free space with a nearby support plane.	Standing position.	Multimodal objects of interest to observe.	Present an object of motivational interest for the child to observe while maintaining the standing position.<br>Facilitate the maintenance of the position with the pleasant interaction of the caregiver, such as singing short songs and rhymes.
	III	Not achievable at this age.	-	-	-	-
	IV-V	Not achievable.	-	-	-	-
**Coastal navigation** (11 months)	I-II	Move laterally from the standing position by leaning on a surface.	Table, support bar attached to the edge of the table.	Standing position with an anterior support surface.	Age-appropriate objects.<br>Table.<br>Support bar.<br>High contrast striped lines on the support plane to enhance the perception of lateral movement in space.	Activate a multimodal toy placed laterally on the table at a distance of 10–30 cm.<br>If needed, guide the hands toward the bar to grasp it.<br>If necessary, overlay a panel with high contrast striped lines on the support plane.<br>If needed, stabilize the pelvis and guide the child in the movement of abduction and adduction of the lower limbs.
	III			Not achievable at this age.<br>When support reactions to load emerge, standing with minimal physical guidance from the operator to initiate movement.		
	IV-V	Not achievable.	-	-	-	-
**Rotations and transition**	I-II	Move/rotate from the standing position by	Two closely placed surfaces positioned to	Standing position with 90° rotation.	Age-appropriate objects.	Place the object on the farther surface or move it to motivate the child to reach it by

**between support surfaces** (11–12 months)	III	90° shifting hands for support.	create a 90° angle.	Not achievable at this age. When load support reactions emerge, standing position with 45° rotation.	(interlocking toys, building blocks, books, and everyday objects). Support surfaces. Different high-contrast surfaces.	transitioning between surfaces. To facilitate the rotation of the pelvis toward the other surface, stabilize and slightly support it. To help with visual perception of the different spaces to reach, overlay panels with high contrast patterns (such as checkered boards with varying size squares or striped lines) on the support surfaces.
	IV-V	Not achievable.	-	-	-	-
	I-II	Move rotating from standing by 90° without maintaining support during the transition with load shift.	Two closely positioned surfaces creating a 90° angle.	Standing position with 90° rotation.	Age-appropriate objects. (interlocking toys, building blocks, books, and everyday objects). Support surfaces. High-contrast surfaces.	Place the object on the farther surface or move it to motivate the child to reach it. To facilitate the load shift, use the pelvic grip and encourage abduction/adduction of the lower limbs. To enhance visual perception of different spaces to reach, overlay high-contrast panels (checkered boards with varying square sizes or striped lines) on the support surfaces.
	III			Not achievable at this age. When load support reactions emerge, standing position with 45° rotation.		
	IV-V	Not achievable.	-	-	-	-
**Walking with support** (11 - 12 months)	I-II	Moving forward by placing the upper limbs on a support (pushcart, stool, chair, adult support). The child can bear their body weight	Table or small bench positioned anteriorly in an unobstructed space.	Standing position with a mobile frontal support (e.g., first steps).	Age-appropriate objects (chair, small bench, table, first steps pushcart). High-contrast panels to overlay on the floor under	Place the object of interest or the caregiver 3 meters away to motivate the child to reach the caregiver or the object. To facilitate the load shift and forward progression, use the pelvic grip to stabilize and/or guide the child in the forward propulsive movement. Move the
	III			Not achievable at this age. When load support reactions emerge, standing position		

		but cannot maintain balance during the movement.		with semi-mobile frontal support (e.g., weighted pushcart).	each foot.	support surface under the foot that needs to lift off the ground to facilitate hip extension toward the posterior space, followed by a flexion response and forward progression of the limb.
	IV-V	Not achievable.	-	-	-	-
**Squatting position and standing up without support** (12 months)	I-II	Bend the lower limbs to reach the object and then return to the standing position without support.	In an open space with some materials placed on the floor.	Squatting position.	Age-appropriate toys.	Place the object of interest on the floor near the child and, if necessary, ask them to lift the object from the ground.<br>To facilitate hip and knee extension, use the pelvic grip.<br>Set up play routines that encourage inserting objects into a container positioned slightly higher than the child's height.
	III			Not achievable at this age. When load support reactions emerge, squatting position with anterior support.		
	IV-V	Not achievable.	-	-	-	-
**Independent walking** (13-14 months)	I-II	Walking characterized by a wide base of support, arms in a high guard position, absence of pendulum movements, full foot contact (absence of heel contact), digitigrade walking.	Open space.	Standing position while moving.	Age-appropriate toys that motivate and interest the child to move towards them (bubbles, balloons, cause-effect toys, sound toys).	Place the object of interest or the caregiver at a distance of 3 meters to motivate the child to reach it.<br>To facilitate weight shifting and progression in space, use the pelvic grip to support the child's movement through the flexion-extension of the lower limbs.<br>To help the child perceive the space for movement, high-contrast zebra-striped panels can be placed along the path to guide the child.
	III			Not achievable at this age. When load-bearing reactions emerge, standing position, with initial input from the operator.		

	IV-V	Not achievable.	-	-	-	-
**Autonomous walking with direction changes** (14-24 months)	I-II	Autonomous walking (with or without aids) within a room with some direction changes.	Open space.	Standing position while moving.	Age-appropriate toys that motivate and engage the child to move toward them in different positions within the space.	Set up the room with multiple shelves at the child's height, placed at a distance from each other, where objects and toys of interest are placed, allowing the child to reach them freely.
	III			Not achievable at this age. When load-bearing reactions emerge, standing position, with initial input from the operator.		
	IV-V	Not achievable.	-	-	-	-
**Autonomous walking with transporting objects and toys** (14-24 months)	I-II	Autonomous walking (with or without aids) within a room while simultaneously transporting objects.	Open space.	Standing position while moving.	Age-appropriate toys that motivate and engage the child to move, transport, and deliver them within the room.	Arrange the room so that the child is encouraged to reach objects of interest that are accessible for them to pick up, with the goal of transporting them to engage in a motivating play with the caregiver.
	III			Not achievable at this age. When load-bearing reactions emerge, standing position, with initial input from the operator.		
	IV-V	Not achievable.	-	-	-	-
