# Supplementary material for: A Multidimensional and Integrated Rehabilitation Approach (A.M.I.R.A.) for Infants at Risk of Cerebral Palsy and Other Neurodevelopmental Disabilities
Source: Children (Basel). 2025 Jul 30;12(8):1003. doi: 10.3390/children12081003 (PMC12384761; doi:10.3390/children12081003)
Supplement: Supplementary file 1 [file children-12-01003-s001.zip › Table S5 - Manipulative-Praxic Function Chart.pdf]

Table S5 - Manipulative-Praxic Function Chart

Premises for using the chart

- All the proposals described below refer to a rehabilitative approach that considers the child in their entirety, that is, as a mind-body unit. According to this perspective, all the functions are closely interconnected and are organized to cooperate with each other in order to achieve a specific goal, aimed at the child's optimal adaptation to the surrounding environment. When cooperation between multiple functions is not possible or is difficult, and the optimal adaptation of the child to the living environment cannot be achieved, the characteristics of the environment must be adapted to the child's needs and requirements through perceptual-motor facilitation interventions.
- The proposals include an initial phase of observation of the child's attempts to actively experiment with autonomous action strategies. From observing the difficulties the child faces during these attempts, a "facilitating" phase follows, consisting of perceptual-motor guidance to action, which should enable the child to succeed in the actions outlined by the specific proposal. Once the child has mastered the specific skill through facilitating intervention, they are allowed to actively and autonomously experiment with the specific actions prescribed in the proposal, so that, through trial and error, they can select the most effective strategies to achieve their goal. Only after this can the proposal be gradually modified in a "challenging" direction, by progressively adding levels of complexity and increasing difficulty. The increase in the level of challenge can be achieved by modifying the demands, reducing the facilitations used, and requiring the simultaneous control of multiple functions during the same task.
- Proposals that are effective in producing an adaptive change in the child during therapy should be shared with the family, working together to find strategies for transferring them to the home environment. Family members should be supported in understanding the goals of the various proposals, in paying attention to the child's reactions, and in managing the timing of the proposals (e.g., when during the day, in which daily life situations, for how long, how many times a day, etc.).
- The selection of objects and activities, as well as the adaptation of the context (from the options indicated in the table), are variable and depend on the child's functional level, following the indications provided by the classification scales (VFCS; GMFCS, Mini-MACS). The choice of the direction of the proposal, whether facilitative or challenging, the duration and number of proposals, and the time to be dedicated to each individual proposal within the rehabilitative plan must necessarily vary from child to child and, for the same child, from session to session, depending on their interest, needs, motivation levels, and availability, in order to support their motivation and enjoyment of learning.
- In the presence of visual engagement difficulties in the child, it is recommended to evaluate the opportunity of using a chessboard and/or high-contrast black-and-white images and objects that can amplify the visual perception cues related to the objects in use and the child's action context. These precautions help facilitate the child's

attentional orientation, enabling the integration of information from the visual channel with the other functions. The chessboard can be used alone as an attentional cue or as a background to objects, amplifying the perception between objects and background. Another useful precaution is to provide soft lighting in the room (free from direct and intense light sources) and use a flashlight to illuminate the child's or caregiver's face, or the objects being proposed.

- When proposing multisensory objects, take the child's characteristics into account, prioritizing the sensory channel to which they are most sensitive (facilitative approach): for example, to promote the grasping of an object, it is possible to gently touch the back of the child's hand to stimulate activation (tactile facilitation) or use a sound-producing object (auditory facilitation).
- If the child is unable to reach and grasp an object, it is helpful to modify the object to make it "graspable" and, if necessary, guide the movement, allowing the child to repeat the action multiple times in stable conditions before changing the object or task. This facilitates understanding of the movement rules and subsequent behavioral mastery in performing it.
- If a decline in attention and availability is observed in the child, it is useful to introduce novel elements to regain their attention. This can be achieved by alternating the use of objects (from those described) or using them in combination (e.g., face + flashlight; rattle + chessboard + flashlight, and so on).
- It is useful to schedule rest breaks and change activities when the child shows no further interest in the ongoing activity.
- The overall duration of the proposed activity is related to the child's achievement of the objective and their motivation to continue pursuing it.
- The choice of position that the child can maintain in each specific task is related to their motor skills and must always allow them to perform optimally, especially when multiple functions are involved simultaneously. The reference criterion for determining the appropriate level of challenge during the activity is the "optimal challenge," which provides the child with a reasonable expectation of success. For tasks related to manipulative-practical functions, it is advisable to adopt a facilitating approach for postural control demands to allow the child to direct their resources effectively toward upper limb activities.
- The selection of objects and activities and the adaptation of the context (from the options provided in the table) are variable and depend on the child's functional level, as indicated by the classification scales (VFCS, GMFCS, Mini-MACS), as well as the child's behavior during the session, taking into account their experiences and reactions.
- From the age of one year, the objectives of each activity have been divided and differentiated based on functional levels according to the Mini-MACS classification scales. For some abilities at level V, it was not possible to identify a reachable objective, or an alternative goal was proposed for that ability.
- The use of orthoses, aids, splints, and taping must be evaluated on a case-by-case basis and introduced, if necessary, as an integral part of the rehabilitation project as a facilitating measure.
- The age division is indicative, and it is possible, for each age group, to introduce activities and objects described in previous age groups.

Objectives for Manipulative-Praxic Functions

- Eye-hand and eye-hand-mouth coordination
- Grasping (stabilizing posture to reach and grasp objects, reaching, taking, holding, releasing, throwing, handing over, etc.)
- Adapting the grip to different objects (differentiated grasping)
- Passing the object from one hand to the other, exploring it
- Visual-tactile exploration of objects
- Ability to place various objects according to their nature
- Anticipatory ability (preadaptation) based on previous manipulative experiences
- Finalization of the achieved skill in everyday life situations and its transferability to the child's various life contexts

Age appropriate tools

**0-6 months**	**6-12 months**	**12-24 months**	**Contextual elements**
Checkerboard	Radiating structure	Adapted puzzles with increasing complexity	Mat
Flashlight	Rotating plate	Building blocks	Lighting
Human face	Cubes (1 cm, 2 cm)	Everyday objects (plate, spoon, fork, cup, small pot, brush)	Emotionally significant familiar item
Sound bracelets	Containers	Books with flaps	Soft containment roller
Easter egg card	Rattles	Animals (toys or figurines)	Wedge
Necklaces	Soft books	Toy cars	Roller
Graspable ball	Button-activated toys	Wind-up cars	Soft climbing steps
Soft ball	Spinning top	Push-along walker	Cube or table (height 40 cm)
Sensory ball	Musical instruments	Paper and markers	Checkerboard

	(keyboard, drum, maracas, rattles, rain stick)		
Jingle bells	Graspable objects and toys	Velcro fruit toys	Oscillating platform
Spring	Objects for bimanual manipulation	Rings	Sensory surfaces
Koosh ball	Multimodal toys	Pull-along toy with a string	Black-and-white striped surface
Sound ring	Napkin for object hiding	Images and photos of everyday objects	
Ribbons with jingle bells	Containers and small bottles for pouring activities	Small roller (15 cm in diameter)	Music, songs, nursery rhymes
	Velcro toys		

Manipulative-Praxic Function chart

**0-3 months**						
**Ability**	**MACS**	**Objective**	**Context**	**Child**	**Tools**	**Proposals**
**Organization on the midline**	NA	1. Exploration of the hands with gaze<br>2. Exploration of the hands with gaze and mouth<br>3. Touching hands	Quiet environment, with adapted lighting, free from distracting or confusing factors	1) In the parent's arms, contained position with the caregiver facing the child.<br>2) Supine, inclined about 30° with the head supported by the adult's hand (open palm), contained distally (feet	Age-appropriate objects with graspable features.	Hold the child's hands and move them in front of their eyes, encouraging visual and oral exploration.<br>Present the caregiver's face along the midline and bring the caregiver's hands to it.<br>Facilitation: place high-contrast sound bracelets on the child’s

				resting against the adult's abdomen); positions involving flexion and containment are always preferable.		wrists and support active intermodal exploration (visual, tactile, kinesthetic, oral, auditory).
**Reaching**	NA	1. Evoking a movement of a limb towards the target located on the midline<br>2. Bringing the hand into contact with the target	Quiet environment, with adapted lighting, free from distracting or confusing factors	1) In the parent's arms, contained position with the caregiver facing the child.<br>2) In the bouncer or nest.	Age-appropriate objects with graspable features.<br><br>Checkerboard.	Present objects within the child's visual field, gently brush their hand, and wait for the child to attempt to reach before moving the objects to expand their range of action.<br>Facilitations: Select soft, graspable objects (those that the child can easily hold by opening just a few fingers), such as: necklaces, colored ribbons with attached bells, colorful dolls with long, soft limbs, or simply add ribbons to objects the child is already using but cannot grasp.<br>If the child tends to exclude certain spatial sectors in attention and limb orientation, shield the part of the visual field to which the child preferentially attends, and enrich the ignored or excluded sector by overlaying interesting objects on the checkerboard.
**3-6 months**						
**Ability**	**MACS**	**Objective**	**Context**	**Child**	**Tools**	**Proposals**

**Reaching (rake approach)**	NA	1. Elicit a limb movement toward a target placed at various spatial locations.<br>2. Bring the hand into contact with the target.	Quiet environment, with adapted lighting, free from distracting or confusing factors	In the caregiver's arms, supine, lateral decubitus, prone, or seated with containment.	Age-appropriate objects with graspable features.<br><br>Checkerboard.	Present objects within the child's visual field, gently brush the hand, and wait for the child to attempt reaching before moving the objects to expand the action space.<br><br>Facilitations: If the child tends to exclude spatial sectors in attentional or limb orientation, shield the visual field segment they preferentially attend to and enrich the ignored or excluded sector using a checkerboard with overlaid objects of interest.<br>If asymmetry in upper limb use is present, encourage the use of the less engaged limb by presenting targets more frequently on that side and promoting limb exploration. Optionally, limit the preferred limb by covering it with a small sock.
**Grasping (palmar grasp)**	NA	1. Grasp and hold an object.<br>2. Grasp and hold an object located in the peripersonal space.	Quiet environment, with adapted lighting, free from distracting or confusing factors	In the caregiver's arms, supine, lateral decubitus, prone, or seated with containment.	Age-appropriate objects with graspable features, varying in size, shape, and texture.<br><br>Checkerboard.	Present objects within the child's visual field, gently brush their hand, and wait for attempts at reaching, grasping, and hand-to-mouth activities.<br><br>Facilitation: place a checkerboard behind the object as a background to support visual tracking of the action

						with the object.
**6-12 months**						
**Ability**	**MACS**	**Objective**	**Context**	**Child**	**Tools**	**Proposals**
**Parabolic and direct reaching**	NA	1. Expand the action cones of the upper limbs during reaching tasks.<br>2. Integrate visual and tactile-kinesthetic channels by maintaining gaze on one's hands while manipulating different objects.	Quiet environment, with adapted lighting, free from distracting or confusing factors	Supported sitting, seated on the mat, or seated on a small bench.<br>In a postural system with a table with a recessed opening.	Age-appropriate objects with graspable features, varying in size, shape, and texture.<br><br>Checkerboard.	Present one object at a time within the child's visual field, waiting for them to attempt reaching, grasping, manipulating, and hand-to-mouth activities. Verbally guide the child in exploring the different physical characteristics of the toy (size, shape, texture, color, structure, surface).<br><br>Facilitations: place a checkerboard behind the object as a background to support visual tracking of the action with the object.<br>If the child shows no attempts at grasping, propose multimodal objects suited to the child's characteristics and gently brush their hand to encourage activation.
**Grasping (grip adaptation)**	NA	Promote hand adaptation after grasping the proposed object.	Quiet environment, with adapted lighting, free from distracting or confusing factors.	Supported sitting, seated on the mat, or seated on a small bench.<br>In a postural system with a table with a recessed opening.	Objects and toys of varying size, shape, and texture.	Guide the child's hands in reaching for and grasping the object.<br>Repeat the action multiple times with the same object to help the child achieve behavioral

						mastery before introducing a new object.<br><br>Facilitations: adapt the spatial orientation of the object.
**Grasping (pre-adaptation of grip)**	NA	Facilitate hand and finger orientation before grasping the proposed object.	Quiet environment, with adapted lighting, free from distracting or confusing factors	Supported sitting, seated on the mat, or seated on a small bench.<br>In a postural system with a table with a recessed opening.	Objects and toys of varying size, shape, and texture.	Present a single object at a time, offered in different orientations.<br><br>Facilitations: present the object consistently with the same spatial orientation, allowing the child time to adapt without changing the orientation.
**Bimanual grasping**	NA	Use of both hands to grasp and manipulate a single object or to grasp two separate objects (one in each hand).	Quiet environment, with adapted lighting, free from distracting or confusing factors	Supported sitting, seated on the mat, or seated on a small bench.<br>In a postural system with a table with a recessed opening.	Age-appropriate objects requiring bimanual integration.	Present large-sized objects that require both hands to grasp. Offer two objects to be grasped (one in each hand) and demonstrate simple actions such as shaking or banging them together.
**Grasping (Fine grip) and release**	NA	Promote the grasping of small-sized objects with the following grips:<br>1. Three-finger grasp<br>2- Inferior pincer grasp<br>3. Superior pincer grasp<br>And the release onto a surface.	Quiet environment, with adapted lighting, free from distracting or confusing factors	Supported sitting, seated on the mat, or seated on a small bench.<br>In a postural system with a table with a recessed opening.	Small-sized objects of various shapes (cereal balls, cubes).	Present objects with the described characteristics and encourage grasping, manipulation, and release in activities such as pouring (placing items into a small bottle/container).
**Bimanual manipulation**	NA	Use both hands to grasp and manipulate objects, even with differentiated	Quiet environment, with adapted lighting, free from distracting or	Supported sitting, seated on the mat, or seated on a small bench.	Age-appropriate objects requiring bimanual integration.	Present objects composed of multiple parts that can be attached and detached (e.g.,

		tasks for each limb.	confusing factors	In a postural system with a table with a recessed opening.		building blocks, velcro fruit, markers).<br>Offer objects that encourage differentiated use of the upper limbs (e.g., one hand holds and the other explores).
**Manipulation: functional exploration**	NA	Use both hands to grasp and manipulate objects in order to explore their functional characteristics.	Quiet environment, with adapted lighting, free from distracting or confusing factors	Supported sitting, seated on the mat, or seated on a small bench.<br>In a postural system with a table with a recessed opening.	Toys and everyday objects (e.g., containers, cubes, tableware).	Present objects, allowing the child to experiment with their potential uses, including functional aspects (e.g., opening/closing, stacking, functional use of everyday objects).
**Grasping (Pre-adaptation of the limb and grip)**	NA	Encourage pronation-supination of the forearm and fine grasping.	Quiet environment, with adapted lighting, free from distracting or confusing factors	In the caregiver's arms.<br>In a postural system with a table with a recessed opening.<br>Seated on the mat.	Age-appropriate objects with graspable features.	Present sticks to be held vertically with one hand, while the other attempts to slide rings onto them without letting them slip.<br>Offer simple puzzles or inserts.<br>Present containers and objects for pouring activities.<br>Present books with flaps to open and close, and images to explore.<br><br>Facilitation: if necessary, guide the child's hands in the use of the proposed objects.
**12-18 months**						
**Ability**	**MACS**	**Objective**	**Context**	**Child**	**Tools**	**Proposals**

**Bimanual manipulation**	I-II	Use both hands to manipulate objects, even with differentiated tasks for each limb.	Quiet environment, with adapted lighting, free from distracting or confusing factors	Supported sitting, seated on the mat, or seated on a small bench.<br>In a postural system with a table with a recessed opening.	Age-appropriate objects requiring bimanual integration.	Present objects composed of multiple parts that can be attached and detached (e.g., building blocks, velcro fruit, markers) and/or objects that encourage differentiated use of the upper limbs (e.g., one hand holds and the other explores).
	III-IV	Use compensatory strategies for grasping (e.g., using parts of the body to secure/stabilize the object).				Present objects composed of two parts that can be attached and detached (e.g., comb-like construction toys, velcro fruit, markers), encouraging the discovery of more functional compensatory strategies.
	V	Not-reachable	-	-	-	-
**Releasing**	I-II	Encourage release:<br>1. On the table<br>2. In a large container<br>3. In a small container	Quiet environment, with adapted lighting, free from distracting or confusing factors	Supported sitting, seated on the mat, or seated on a small bench.<br>In a postural system with a table with a recessed opening.	Age-appropriate objects.	Present objects for pouring (e.g., pebbles into a small bottle/container, simple puzzles, stacking cubes).
	III-IV	1. Encourage release on the table or in a large container<br>2. Encourage the discovery of compensatory strategies (e.g., removing the object with the other hand).			Objects that are easy to hold and release (to be selected based on the child's characteristics).	Present objects for pouring (e.g., pebbles) from one tin box to another in a way that produces a motivating effect. Present the basket game with small, soft balls into a large basket.<br><br>Facilitation: gently touch the back of the hand to encourage finger opening, and use an

						elastic-compressive orthosis or functional bandaging.
	V	Not-reachable	-	-	-	-
**18-24 months**						
**Ability**	**MACS**	**Objective**	**Context**	**Child**	**Tools**	**Proposals**
**Intransitive praxis (gestures)**	I-II	Learning simple communicative gestures	Quiet environment, with adapted lighting, free from distracting or confusing factors	Seated with support, seated on the mat, seated on a small bench, or in a posture system with a table with a recessed opening.	None.	Elicit gestures contextually (e.g., waving "goodbye" when leaving, hand to mouth for eating, the drinking gesture if thirsty, "stop" or "go" gestures when the child wants to stop or start an activity, etc.).<br>Present one gesture at a time, accompanied by simple mimed nursery rhymes. After a few demonstrations, stop just before the conclusion and wait for the child to try completing the gesture or movement themselves. Repeat multiple times to promote the automation of the gesture.<br><br>Facilitation: if needed, guide the child's hands.
	III-IV					
	V					
**Transitive praxis**	I-II	Learn simple praxises of daily life	Quiet environment, with adapted lighting, free from distracting or confusing factors	Seated with support, seated on the mat, seated on a small bench, or in a posture system with a table with a recessed opening.	Everyday objects.	Present one object at a time to the child, demonstrating its use and action patterns, repeating the action several times. Leave the object available and wait for

						the child to initiate use without forcing the start (e.g., using a spoon to stir, bringing it to the mouth, combing the doll, etc.).
	III-IV				Adapted everyday objects.	Present one object at a time to the child, demonstrating its use and action patterns, repeating the action several times. Leave the object available and wait for the child to initiate its use without forcing the start.
	V	-	-	-	-	-
**Daily life autonomies**	I-II	Learn simple daily life activities	Quiet environment, with adapted lighting, free from distracting or confusing factors	Seated with support, seated on the mat, seated on a small bench, or in a posture system with a table with a recessed opening.	Everyday objects.	Promote the acquisition of simple actions (e.g., drinking from a cup/glass, using a spoon to eat, washing hands, taking off socks, etc.) in play contexts through imitation and physical guidance (see "autonomy and self-regulation table").
	III-IV				Adapted everyday objects.	
	V	-	-	-	-	-
