# Supplementary material for: A Multidimensional and Integrated Rehabilitation Approach (A.M.I.R.A.) for Infants at Risk of Cerebral Palsy and Other Neurodevelopmental Disabilities
Source: Children (Basel). 2025 Jul 30;12(8):1003. doi: 10.3390/children12081003 (PMC12384761; doi:10.3390/children12081003)
Supplement: Supplementary file 1 [file children-12-01003-s001.zip › Table S6 - Interaction-Communication Function Chart.pdf]

Table S6 - Interaction-Communication Function Chart

Premises for using the chart

- All the proposals described below refer to a rehabilitative approach that considers the child in their entirety, that is, as a mind-body unit. According to this perspective, all the functions are closely interconnected and are organized to cooperate with each other in order to achieve a specific goal, aimed at the child's optimal adaptation to the surrounding environment. When cooperation between multiple functions is not possible or is difficult, and the optimal adaptation of the child to the living environment cannot be achieved, the characteristics of the environment must be adapted to the child's needs and requirements through perceptual-motor facilitation interventions.
- Proposals that are effective in producing an adaptive change in the child during therapy should be shared with the family, working together to find strategies for transferring them into the home environment. Families should be supported in understanding the objectives of the various proposals, in paying attention to the child's reactions, and in managing the timing of the proposals (e.g., when during the day, in which daily life situation, for how long, how many times per day, etc.).
- The selection of objects, activities, as well as the adaptation of the context (among the possibilities indicated in the table) are variable and depend on the child's functional level, following the indications provided by the classification scales (VFCS, GMFCS, Mini-MACS). The choice of the direction of the proposal, whether facilitative or challenging, the duration and number of proposals, and the time to be dedicated to each individual proposal within the rehabilitative plan must necessarily vary from child to child and, for the same child, from session to session, depending on their interest, needs, motivation levels, and availability, in order to support their motivation and enjoyment of learning.
- In the presence of visual engagement difficulties in the child, it is recommended to evaluate the opportunity of using a chessboard and/or high-contrast black-and-white images and objects that can amplify the visual perception cues related to the objects in use and the child's action context. These precautions help facilitate the child's attentional orientation, enabling the integration of information from the visual channel with the other functions. The chessboard can be used alone as an attentional cue or as a background to objects, amplifying the perception between objects and background. Another useful precaution is to provide soft lighting in the room (free from direct and intense light sources) and use a flashlight to illuminate the child's or caregiver's face, or the objects being proposed.
- It is beneficial to involve multiple sensory channels (visual contact, listening, touch, etc.) simultaneously in the interaction proposals to increase motivation for interactive exchange, adjusting them for quantity and intensity based on the child's characteristics and reactions.

- The selection of objects and activities and the adaptation of the context (from the options provided in the table) are variable and depend on the child's functional level, as indicated by the classification scales (VFCS, GMFCS, Mini-MACS), as well as the child's behavior during the session, taking into account their experiences and reactions.
- If a decline in attention and availability is observed in the child, it is useful to introduce novel elements to regain their attention. This can be achieved by alternating the use of objects (from those described) or using them in combination (e.g., face + flashlight; rattle + chessboard + flashlight, and so on).
- The overall duration of the proposed activity is related to the child's achievement of the objective and their motivation to continue pursuing it, with alternating periods of activity and moments for rest and recovery of well-being.
- The age division is indicative, and it is possible, for each age group, to introduce activities and objects described in previous age groups.

Objectives for Interaction-Communication Functions

- Perception, orientation to sounds, noises, voice
- Recognition of the voice
- Focal attention to communication
- Communicative and intentional responses (verbal and non-verbal)
- Socially motivated actions and imitative behavior (e.g., saying goodbye, pointing, pretending to eat, drink, feeding the doll, etc.)
- Verbal comprehension of simple familiar words and everyday phrases
- Non-verbal communication: grimace and smile, communicative gestures, pointing, showing
- Verbal communication: types of crying, vocalizations, babbling, simple words, and short sentences

Age appropriate tools

**0-6 months**	**6-12 months**	**12-24 months**	**Contextual elements**
Human face	Human face	Human face	Mat
Flashlight	Flashlight	Flashlight	Lighting
Checkerboard	Checkerboard	Books with images that inspire onomatopoeic sounds	Affectionately significant family member

Multimodal toys and rattles	Multimodal toys and rattles	Nursery rhymes and songs	Soft containment rolls
Bells	Nursery rhymes and songs	Gestures, facial expressions, sounds to imitate	Armchair, booster seat

Interaction-Communication Function chart

**0-6 months**					
**Ability**	**Objective**	**Context**	**Child**	**Tools**	**Proposals**
**Pre-verbal communication**	Eye contact and visual interaction: The child<br>1. Looks at a person.<br>2. Recognizes the mother and follows her when she moves.<br>3. Exhibits a social smile.<br>4. Orients toward the mother's voice.<br>5. Vocalizes in response to the mother's voice.<br>6. Vocalizes in response to strangers.<br>7. Calms down with the mother's voice (please refer to objectives for self-regulation functions).	Calm environment free from distracting or confusing factors, close distance (20–30 cm) with appropriately adapted lighting.	In the parent's arms, facing each other.<br><br>Seated in a car seat, reclining chair, or postural system (in a contained space where the child is calm).<br><br>Seated on the floor mat.<br><br>Child in a state of calm wakefulness.	Human face.<br><br>Caregivers.<br><br>Use of multisensory/motivating materials.	Position oneself in front of the child, illuminating the face if necessary, and adjust prosody (warm, slow, and welcoming or lively and stimulating) according to the child's activation threshold. Wait for the child to establish visual contact with the interlocutor's face, then aim to maintain eye contact. Calibrate interactive proposals with pauses, allowing time for recovery and reactivation from the child. Emphasize the presence of the individual using multisensory cues that are engaging for the child and place the object at the level of their face. Slowly move the object to promote orientation actions.

**Pre-verbal interaction and communication**<br><br>Response to social cues.<br>Activation of intersubjective behaviors in reaction to the social cues of others.	1. Vocalizations, eye contact, imitation of facial expressions, smile, gestures.<br>2. Joint emotion / shared enjoyment.	Calm environment free from distracting or confusing factors, close distance (20–30 cm) with appropriately adapted lighting.	In the parent's arms, facing each other.<br><br>Seated in a car seat, reclining chair, or postural system (in a contained space where the child is calm).<br><br>Seated on the floor mat.<br><br>Child in a state of calm wakefulness.	Caregivers.<br><br>Age-appropriate objects.<br><br>Socio-sensory play routines with or without objects.	Entertain the child with stable and enjoyable interactive play routines, including songs, gestures, and facial expressions. After several repetitions of the same routine, in a challenging manner, interrupt the sequence midway and wait for any response from the child (gesture, movement, vocalization, eye contact) that indicates an attempt to reactivate the play proposal by the adult.
**6-12 months**					
**Ability**	**Objective**	**Context**	**Child**	**Tools**	**Proposals**
**Pre-verbal interaction and communication**<br><br>Activation of intersubjective behaviors to initiate and maintain joint attention.<br>Initiative.	1. The child looks/extends arms/smiles to request the repetition of a routine.<br>2. The child hands over/extends an arm/points to initiate a routine.<br>3. Triangulation.<br><br>Response behaviors in the presence of an object:<br>The child<br>1. Follows the adult's indication in	Calm environment free from distracting or confusing factors, with appropriately adapted lighting.<br><br>Adult in the child's peripersonal space.<br><br>Adult in the child's frontal space.	In the parent's arms, facing each other.<br><br>Seated on the floor mat.<br><br>Seated in a car seat, reclining chair, or postural system.	Caregivers.<br><br>Age-appropriate objects.<br><br>Socio-sensory play routines with or without objects.<br><br>Use of multisensory objects, prioritizing the sensory characteristics preferred by the child.	Present a motivationally engaging object to the child, allowing them to interact freely while maintaining an active availability to respond to their attempts at sharing (seeking eye contact, enthusiastically commenting on their interaction with the object, providing verbal reinforcement).<br><br>Alternate the adult's emphasis with pauses, modulating the timing according to the child's activation levels, gradually increasing (challenging sense) to promote extended attention spans.<br><br>Present an object outside the child's peripersonal space, adjusting the distance from initially facilitating to progressively challenging. The object is activated by the examiner, and when it is turned off, wait for the child to seek the adult's gaze as a request for reactivation. The response latency of

	the proximal space and later in the distal space.<br>2. Orient the gaze toward where the adult is looking.				the child can be supported by indicating the object.<br><br>After the child's actions/vocalizations, the adult imitates and mirrors the child's actions and productions.
**Relationship - interaction**	Separation - individuation process.	Calm environment free from distracting or confusing factors, with appropriately adapted lighting.<br><br>Adult in the child's peripersonal and extrapersonal space.<br><br>Adult in the child's frontal space.	The child in the adult's peripersonal space, with the adult remaining within the child's auditory/visual field.	Caregivers.<br><br>Motivating play activities.	In the presence of separation difficulty:<br>Promote the gradual introduction of another adult into the parent-child space, followed by the gradual distancing of the primary caregiver, also through games like peek-a-boo.<br><br>Motivating play activities for the child, shared with the therapist.<br>Gradual distancing of the therapist from the child's play space (remaining within the visual field).
**Preverbal communication**	1. Imitation (see cognitive function objectives).<br>2. Turn-taking.	Calm environment free from distracting or confusing factors, with appropriately adapted lighting.<br><br>Adult in the child's peripersonal space.<br><br>Adult in the child's frontal space.	In the parent's arms, facing each other.<br><br>Seated on the floor mat.<br><br>Seated in a car seat, reclining chair, or postural system.<br><br>Standing.	Caregivers.<br><br>Age-appropriate objects.	Following the child's actions/vocalizations, the therapist imitates/mirrors them.<br><br>Immediate imitation: use two identical objects and wait for the child to repeat the observed action.<br><br>Turn-taking: show the child some play actions using sound-producing objects activated by buttons, switches, or a charge; make the toy available to the child, visually facilitating the transfer with a slow movement accompanied by comments or simple words. Repeat the turn-taking routine with the object multiple times until it engages and delights the child. Subsequently, interrupt the action and wait for the child to initiate and request the toy again.

	Production/ imitation of coded gestures.	Calm environment free from distracting or confusing factors, with appropriately adapted lighting.<br><br>Adapt the position, e.g., behind the child in front of a mirror, beside them...	In the parent's arms, facing each other.<br><br>Seated on the floor mat.<br><br>Seated in a car seat, reclining chair, or postural system.<br><br>Standing.	Caregivers.	In socio-sensory routines, include simple gestures that the child can imitate (songs, nursery rhymes, waving, peek-a-boo, clapping, etc.), and if necessary, facilitate the child by guiding them in performing the gesture.<br><br>In a challenging manner, pauses can be inserted while waiting for possible reactions from the child, gradually increasing the waiting time before continuing with the song.
**Verbal communication**	Babbling (simple and varied).	Calm environment free from distracting or confusing factors, with appropriately adapted lighting.	Seated at a table.<br><br>On the floor mat.	Caregivers.	In a playful and enjoyable interactive context, encourage the emergence of babbling by introducing high-frequency words (e.g., "ma-ma"). Ensure eye contact is established and emphasize the movements of the oro-buccal region required for producing the specific sound. Present combinations of syllables paired with simple, repetitive gestures (e.g., clapping).
**12-24 month**					
**Ability**	**Objective**	**Context**	**Child**	**Tools**	**Proposals**
**Preverbal communication**	Requestive deixis:<br>1. Close-range pointing.<br>2. Distant pointing.	Calm environment free from distracting or confusing factors, with appropriately adapted lighting.<br><br>The practitioner remains within the child's peripersonal space.	Seated at a table.<br><br>On the floor mat.	Caregivers.<br><br>Age-appropriate objects.	Place an object of interest on a surface or in a visible but inaccessible space, prompting the child to use pointing as a request tool by modeling the gesture. Facilitate the child’s attempt by presenting the object within their peripersonal space and/or manually guiding their hand to perform the pointing gesture. Assist in refining the action if the child reaches with their entire hand, and gradually increase the complexity by presenting the object in extrapersonal space and removing physical guidance. Offer the child a choice between two objects or conditions to further

					encourage selective pointing.
	Declarative deixis: a gesture to share interest with the interlocutor for a contextual event.	Calm environment free from distracting or confusing factors, with appropriately adapted lighting.<br><br>The practitioner remains within the child's peripersonal space.	Seated at a table.<br><br>On the floor mat.	Caregivers.<br><br>Age-appropriate objects.<br><br>Containers or screens to hide objects.<br><br>Books.	Provide a model of declarative gesture within a motivating play routine (e.g., pointing to a flying balloon, a fallen car, or a passing train outside).<br><br>One operator, acting as the child's ally, hides an object of interest under a screen. A second operator theatrically searches for the object, asking the child for guidance while the first operator verbally and physically assists the child in pointing to the screen where the hidden object is located.
	Referential gestures (blowing a kiss, "good!", "bye", "it's gone", "hello", "drive"...).	Calm environment free from distracting or confusing factors, with appropriately adapted lighting.<br><br>The practitioner remains within the child's peripersonal space.	Seated at a table.<br><br>On the floor mat.	Caregivers.<br><br>Age-appropriate and motivating objects.<br><br>Books.	The therapist proposes, supports, and emphasizes the production of referential gestures during shared reading, songs, and games with the caregiver.<br><br>A facilitating strategy involves providing the imitative gesture model.
**Verbal communication**	Understanding simple requests.	Calm environment free from distracting or confusing factors, with appropriately adapted lighting.<br><br>The practitioner remains within the child's peripersonal space.	Seated at a table.<br><br>On the floor mat.	Caregivers.<br><br>Age-appropriate objects.	Ask the child to respond to simple commands (e.g., "give me," "hold," "put it in the box..."), supporting the verbal command with a gestural amplification of its meaning (e.g., visibly extending the hand and/or physically guiding the child as facilitating strategies).
	Understanding	Calm environment free	Seated at a table.	Caregivers.	Ask the child to locate high-frequency objects both

	high-frequency vocabulary<br>(choice upon verbal request).	from distracting or confusing factors, with appropriately adapted lighting.<br><br>The practitioner remains within the child's peripersonal space.	On the floor mat.	Age-appropriate objects.<br><br>Containers or screens to hide objects.<br><br>Books.	in spontaneous play conditions and by identifying the requested item among two or more concrete objects presented to them.<br><br>As a facilitating strategy, initially present one high-frequency object and one low-frequency object to ease the correct response.
	Onomatopoeia.	Calm environment free from distracting or confusing factors, with appropriately adapted lighting.<br><br>The practitioner remains within the child's peripersonal space.	Seated at a table.<br><br>On the floor mat.	Caregivers.<br><br>Concrete objects.<br><br>Images.	Associate the presentation of certain objects with the production of corresponding onomatopoeias (e.g., cow – moo; food – yum; etc.) and wait for any attempt at imitation from the child, gradually increasing the waiting time as a challenging approach. Facilitate the child by pairing the sound with gestures.
	First words.	Calm environment free from distracting or confusing factors, with appropriately adapted lighting.<br><br>The practitioner remains within the child's peripersonal space.	Seated at a table.<br><br>On the floor mat.	Caregivers.<br><br>Concrete objects.<br><br>Images and photos of people, objects, and everyday places.	Make sure to be face-to-face with the child while speaking, enunciate the words clearly, repeating them several times with varying tones of voice. Avoid distorting words with diminutives or pet names, and motivate the child with expressive emphasis, rewarding and praising them.<br><br>Comment on the actions during play using single words or combinations of words.
**Relationship - Interaction**	Separation - individuation process.	Calm environment free from distracting or confusing factors, with appropriately adapted lighting.<br><br>The adult in the child's	The child in the room with the adult.	Caregivers.<br><br>Motivating play activities.	In cases of difficulty with separation from the primary caregiver, initially proceed in a facilitative manner and then gradually introduce more challenging elements.<br><br>Support the child by reassuring them verbally and emotionally, assisting them in re-establishing

		peripersonal and extrapersonal space.<br><br>The practitioner in the child’s frontal space.			proximity to the mother when needed. Promote self-soothing behaviors, such as seeking comfort or engaging in motivating activities.<br><br>Create play routines in which the mother leaves the child's visual field (e.g., leaving the room) and then returns, with progressively longer waiting periods.<br><br>In cases of low awareness or indifference toward separation, establish a greeting routine to support the differentiation between the caregiver and the therapist.
