# Supplementary material for: A Multidimensional and Integrated Rehabilitation Approach (A.M.I.R.A.) for Infants at Risk of Cerebral Palsy and Other Neurodevelopmental Disabilities
Source: Children (Basel). 2025 Jul 30;12(8):1003. doi: 10.3390/children12081003 (PMC12384761; doi:10.3390/children12081003)
Supplement: Supplementary file 1 [file children-12-01003-s001.zip › Table S7 - Cognitive Function Chart.pdf]

Table S7 - Cognitive Function Chart

Premises for using the chart

- All the proposals described below refer to a rehabilitative approach that considers the child in its entirety, that is, as a mind-body unit. According to this perspective, all functions are closely connected to each other and are organized each time, cooperating to achieve a specific goal for optimal adaptation of the child to the surrounding environment. If cooperation between multiple functions is not possible or is difficult, and thus optimal adaptation of the child to the living environment cannot be achieved, the characteristics of the environment must be adapted to the child's needs and requirements through perceptual-motor facilitation interventions.
- The proposals include an initial phase of observation of the child's attempts to actively experiment with autonomous action strategies. From observing the difficulties the child faces during these attempts, a "facilitating" phase follows, consisting of perceptual-motor guidance to action, which should enable the child to succeed in the actions outlined by the specific proposal. Once the child has mastered the specific skill through facilitating intervention, they are allowed to actively and autonomously experiment with the specific actions prescribed in the proposal, so that, through trial and error, they can select the most effective strategies to achieve their goal. Only after this can the proposal be gradually modified in a "challenging" direction, by progressively adding levels of complexity and increasing difficulty. The increase in the level of challenge can be achieved by modifying the demands, reducing the facilitations used, and requiring the simultaneous control of multiple functions during the same task.
- Proposals that are effective in producing an adaptive change in the child during therapy should be shared with the family, working together to find strategies for transferring them to the home environment. Family members should be supported in understanding the goals of the various proposals, in paying attention to the child's reactions, and in managing the timing of the proposals (e.g., when during the day, in which daily life situations, for how long, how many times a day, etc.).
- The selection of objects and activities, as well as the adaptation of the context (from the options indicated in the table), are variable and depend on the child's functional level, following the indications provided by the classification scales (VFCS; GMFCS, Mini-MACS). The choice of the direction of the proposal, whether facilitative or challenging, the duration and number of proposals, and the time to be dedicated to each individual proposal within the rehabilitative plan must necessarily vary from child to child and, for the same child, from session to session, depending on their interest, needs, motivation levels, and availability, in order to support their motivation and enjoyment of learning.
- In the presence of visual engagement difficulties in the child, it is recommended to evaluate the opportunity of using a chessboard and/or high-contrast black-and-white images and objects that can amplify the visual perception cues related to the objects in use and the child's action context. These precautions help facilitate the child's attentional orientation, enabling the integration of information from the visual channel with the other functions. The chessboard can be used alone as an attentional cue

or as a background to objects, amplifying the perception between objects and background. Another useful precaution is to provide soft lighting in the room (free from direct and intense light sources) and use a flashlight to illuminate the child's or caregiver's face, or the objects being proposed.

- If a decline in attention and availability is observed in the child, it is useful to introduce novel elements to regain their attention. This can be achieved by alternating the use of objects (from those described) or using them in combination (e.g., face + flashlight; rattle + chessboard + flashlight, and so on).
- It is useful to schedule rest breaks and change activities when the child shows no further interest in the ongoing activity.
- The overall duration of the proposed activity is related to the child's achievement of the objective and their motivation to continue pursuing it.
- The proposals to promote the abilities of this function should stimulate the child's curiosity and motivate them to seek autonomous problem-solving strategies through trial and error, allowing them to extract invariant rules and select effective actions.
- The "cognitive functions" grid was constructed following an evolutionary approach, in steps of learning, based on the progression of the development of abilities in the child.
- In the development of the Table, specific therapeutic goals and proposals were derived using standardized tools as a reference, namely three different developmental scales (see bibliography), from which various items were extrapolated and integrated to define a correct progression of cognitive abilities.
- The age division is indicative, and it is possible, for each age group, to introduce activities and objects described in previous age groups.
- The table specifies whether the proposal is in a facilitating (F) or challenging (C) direction in the columns referring to the context and the proposed activities.

Objectives of Cognitive Function

- Ability to explore the surrounding environment and recognize people, places, and objects
- Progressively more complex organization of play
- Activities with objects (reproduction of simple patterns, differentiation and generalization of patterns, integration of simple goal-oriented patterns, analysis and discovery of variable effects with objects, mental persistence of the object's image, exploration of spatial and causal relationships with objects)
- Ability to predict outcomes and anticipatory adjustment of actions by searching for strategies to solve a problem
- Increase in attention span and differentiation of attentive behaviors in different situations
- Expansion of mnemonic skills
- Self-determination through choice
- Imitation
- Observation and recognition of body parts on oneself and others

- Experimentation and initial organization of body schema
- Adaptation to various social situations (meals, bath, walk, play, etc.)
- Ability to predict outcomes and anticipatory adjustment of actions (involves the ability of mental representation and coincides with the search for strategies to solve a problem)

Age appropriate tools

**0-3 months**	**3-6 months**	**6-12 months**	**12-18 months**	**18-24 months**	**Contextual elements**
High-contrast objects	High-contrast tactile books	Musical instruments (e.g., bells, drum, xylophone)	Small objects to hide	Blocks	Mat or play rug
Auditory objects (e.g., bells)	Tactile sensory objects	Mats	Containers, boxes, fabrics, cups	Toy animals	Adequate lighting
Tactile sensory objects	Light-based sensory objects	Cushions	Proto-symbolic objects (e.g., toy cars, brush, cutlery)	Car track set	Emotionally significant adult
Human face	Human face	Fabrics	Large, soft building blocks	Toy house	Calm environment, free from distracting or confusing factors
Flashlight	Caregiver's body	Objects with diverse sensory features (e.g., luminous, auditory)	Finger paints	Toy cookware	Cube
	Mirror	Cause-and-effect toys (e.g., button-activated, lever-based, sound-producing, pop-up toys)	Shape-sorting box	Doctor's kit	Table
	Flashlight	Books with images (e.g., animals, objects)	Stacking rings	Personal care items	Table with an inset

		Balls	Large sheets of paper/markers	Modeling clay	Music, songs, nursery rhymes
		Flashlight	Books with pictures or flaps	Shaped molds	Sensory surfaces
			Doll	Stickers/adhesive decals	
			Stackable objects	Simple puzzles	
			Pull-string toy	Toy with wheels and pull-string	
			Velcro fruit toys	Wind-up toy cars	
			Images and photos of everyday objects		
			Ball track toy		

Cognitive Function charts

**0-3 months**					
**Ability**	**Objective**	**Context**	**Child**	**Tools**	**Proposals**
**Attention and attentional orientation**	1. Maintains visual attention toward the adult directly in front of them.<br>2. Turns their head toward a person calling them from the side using voice or multisensory objects.	Dim lighting with focused illumination on the caregiver's or therapist's face or objects using a flashlight (F).<br><br>Quiet environment free of distracting or confusing factors (F).	Supine on a mat.<br>In the caregiver's arms.<br>Seated in a small armchair, bouncer, or postural support system.	The caregiver's body and the therapist's face as stimuli.<br><br>Objects with high-contrast colors and multisensory properties (auditory, tactile, and visual).	The child is gently called from the side by an adult positioned behind them and allowed time to turn toward the source of stimulation.<br><br>High-contrast, multisensory objects (e.g., auditory, tactile, visual) or a bell are presented on both hemifields, awaiting an attentional orientation response (F).

		Ecological context not specifically adapted (S).		Face-to-face interaction at an optimal focal distance (approximately 30 cm).	
**Exploratory attention**	1. Visually scans the environment, fixating on objects for approximately 3 seconds.<br>2. Displays an information-oriented response (to auditory and visual stimuli).<br>3. Exhibits facial mimicry in reaction to the approach of the caregiver's or therapist's face.	Adapted lighting.<br><br>Quiet environment free of distracting or confusing factors (F).<br><br>Ecological context not specifically adapted (S).	Supine on a mat.<br>In the caregiver's arms.<br>Seated in a small armchair, bouncer, or postural support system.	The caregiver's body and the therapist's face.<br><br>Objects with high-contrast colors and multisensory properties (auditory, tactile, and visual).<br><br>Face-to-face interaction at an optimal focal distance (approximately 30 cm).	Allows time for free exploration of the room by the child.<br><br>While remaining in the extrapersonal space, the child's attention is gently drawn by producing sounds or noises, or by softly calling them in various spatial sectors (in front, to the right, to the left, in the lower space, and in the upper space).
**3-6 months**					
**Ability**	**Objective**	**Context**	**Child**	**Tools**	**Proposals**
**Focused attention**	1. Continuously considers objects for 5 seconds.<br>2. Shifts attention (visual or auditory) by visually tracking an object that gradually disappears.<br>3. Displays curiosity toward new objects or images compared to	Environment with soft lighting: the caregiver's or therapist's face, objects, or parts of the child's body (F) are illuminated with a flashlight.<br><br>Quiet environment free of distracting or	Supine on a mat.<br>In the caregiver's arms.<br>Seated in a small armchair, bouncer, or postural support system.	The face and body of the caregiver or therapist.<br><br>Objects with various sensory characteristics.<br><br>High-contrast tactile books.<br><br>Mirror.	Face-to-face interactions (F).<br><br>Offering objects in the child's peripersonal space placed in different positions in the surrounding space (C).<br><br>Proposing facial expressions and simple gestures.<br><br>Encourage the child to repeat gestures in front of a mirror, allowing time for observation and the child’s initial attempts at imitation and interaction

	familiar ones.<br>4. Maintains interest in a new activity or person for 30 seconds.<br>5. Shakes a rattle while observing it.	confusing factors (F).<br><br>Ecological context not specifically adapted (S).		Chessboard.<br><br>Flashlight.<br><br>Elastic bands with bells.	with the mirror.<br><br>Barefoot, place elastic bands with bells on one foot at a time, and if helpful, overlay the feet on a high-contrast panel to aid orientation and maintain attention on them (F).
**Distributed attention**	1. Visually explores the environment.<br>2. Shows interest in the mirror.<br>3. Demonstrates awareness of being in a new setting.				
**Interactive play**	Tonic-emotional play: engages interactively with the caregiver through eye contact, smiling, and vocalizing, involving socio-sensory routines.				
**Exploratory play**	Explores the object using vision, touch, or mouth.				
**Body exploration play**	Begins to explore their own body: brings objects and hands to the mouth, observes hands and feet.				
**6-12 months**					

<table>
<tr><th>Ability</th><th>Objective</th><th>Context</th><th>Child</th><th>Tools</th><th>Proposals</th></tr>
<tr><td>Memory</td><td>Distinguishes familiar faces from unfamiliar ones.</td><td rowspan="6">Environment with soft lighting: the caregiver's or therapist's face, objects, or parts of the child's body (F) are illuminated with a flashlight.

Checkerboard on which objects and toys are placed (F).

Quiet environment free of distracting or confusing factors (F).

Ecological context not specifically adapted (S).</td><td rowspan="6">Supine on a mat.
In the caregiver's arms.
Seated in a small armchair, bouncer, or postural support system.</td><td rowspan="6">Age-appropriate objects that activate upon contact (e.g., bells, drum, xylophone).

Use of psychomotor materials (mats, cushions, cloths, supports at various heights).

Objects with different sensory characteristics.

Books with images (e.g., animals, objects).

Cause-and-effect toys (button-operated, lever-based, sound-producing).</td><td rowspan="6">Observe the child's reactions to the face of the caregiver, the therapist, or a stranger.

Once an object or toy of interest to the child is identified, slowly demonstrate the action of partially covering it with a cloth or napkin and wait for the child to act to retrieve it (F). When the child shows the ability to retrieve it, gradually hide it more until it is completely covered (C).

Initially propose the game of peekaboo with a familiar person, paying attention to the child’s reactions and ensuring no fear is elicited (F). Later, propose it with the therapist’s face and wait for the child to respond and repeat the game.

In a shared atmosphere of play and fun, propose an object of interest in various positions within the child’s peripersonal space, in a fixed position.

Offer cause-and-effect games with demonstrations (e.g., activation of button-operated objects, sound/light toys, balls, pop-up toys).

Allow the child to experiment with the most effective ways to reach the motivational object through a trial-and-error approach.</td></tr>
<tr><td>Object permanence</td><td>Searches for a partially hidden object, then a completely hidden object.</td></tr>
<tr><td>Interactive play</td><td>Tonic-emotional play: interest in the game of peekaboo.</td></tr>
<tr><td>Sensory-motor play</td><td>1. Shows interest in active play through the exploration and understanding of their own body in space, as well as sensory manipulation of objects.
2. Propose cause-and-effect games with demonstrations (e.g., activating button-operated objects, sound/light toys, balls, pop-up toys, etc.).</td></tr>
<tr><td>Cause-and-effect play</td><td>Intentionally rings a bell.</td></tr>
<tr><td>Exploratory attention</td><td>Explores the environment and shows</td></tr>
</table>

	increased interest in objects.<br>Demonstrates interest in the images of a book.				
**Exploratory play of cause-and-effect relationship**	Self-knowledge as an agent on the world: the child intentionally strikes objects, pulls, and manipulates them	Quiet environment free of distracting or confusing factors (F).<br><br>Ecological context not specifically adapted (S).<br><br>Table with a recessed opening.	Seated in the postural system or high chair (F).<br><br>On the mat.	Cause-and-effect toys (button-operated, lever-based, sound-producing).<br><br>Age-appropriate objects activated by contact (e.g., bells, drum, xylophone).<br><br>Pull-to-close objects (objects that can be pulled by a string to bring them closer).<br><br>Use of psychomotor materials (mats, cushions, cloths, supports at various heights).<br><br>Objects with various sensory characteristics: items designed to stimulate different senses (e.g., texture, sound, color).<br><br>Books with images (e.g., animals, objects).<br><br>Cause-and-effect toys	Propose cause-and-effect games with demonstrations (e.g., activation of button-operated objects, sound/light toys, balls, pop-up toys) (F).<br><br>In a shared atmosphere of play and fun, offer an object of interest in different areas of the child's peripersonal space, in a fixed position. Allow the child to experiment with the most effective ways to reach the object through a trial-and-error approach.
**Exploratory play of spatial relationships**	1. Develops the spatial relationship between the self and the world, such as searching for a dropped object.<br>2. Explores spatial relationships between objects, for example, pulling items out of a box.				
**Problem solving games**	Perseverance in the task and search for strategies.				

				(button-operated, lever-based, sound-producing).	
**12-18 months**					
**Ability**	**Objective**	**Context**	**Child**	**Tools**	**Proposals**
**Self-determination**	Choosing between two objects	Quiet environment free of distracting or confusing factors (F).<br><br>Ecological context not specifically adapted (S).	Seated in the postural system or high chair.<br><br>Table with a recessed opening (F).<br><br>On the mat (S).<br><br>Seated with a regular table (S).	Age-appropriate objects.	In a shared atmosphere of play and fun, allow the child to choose and reach their preferred toy between two options.<br><br>Give the child time to reach objects and toys by pulling a string or dragging a mat on which the items are placed.<br><br>Provide objects to be inserted into one another, stacked, or placed side by side (C). Verbally describe and explain spatial placements and topological concepts (above/below, inside/outside, near/far, in front/behind) for the child, allowing active exploration of shapes, distances, orientations, directions, and positions of objects (F).
**Exploratory play of spatial relationships**	The child is interested in experimenting with spatial relationships in the environment and contrasts between objects: opening and closing, putting in and out, near and far.				
**Practical-constructive play**	Initial practical-constructive play: stacks a tower of 2 blocks, imitates simple shape fitting (e.g., shape sorters).				
**Functional and protosymbolic play**	The child begins to understand the function of objects and uses them appropriately. Short sequences of imitative play emerge, reflecting scenes observed from familiar figures: combing,			Everyday objects and protosymbolic use (such as cups, toy cars, brushes, utensils, dolls).	Introduce simple protosymbolic play patterns by providing the adult's imitative model (F).<br><br>Propose brief sequences of functional play through imitation (F), first experimenting on oneself and then on another (caregiver, doll), gradually diversifying the play patterns offered (C).

	eating, rocking, driving.				
**Graphic play**	Makes scribbles with a marker.			Finger paints, large markers	In a shared playful context, allow the child to experiment with colors and markers, initially guiding gently the grasp of the color and/or the graphic gesture (F).
**Object permanence**	Finds the hidden object.	Quiet environment free of distracting or confusing factors (F).<br><br>Ecological context not specifically adapted (S).	Seated in the postural system or high chair.<br><br>Table with a recessed opening (F).<br><br>On the mat (S).<br><br>Seated with a regular table (S).	Small objects to hide.<br><br>Cups, boxes, clothes.	In a shared playful context, show the child objects, then hide them, and demonstrate that the object remains, enriching the game with sounds and facial expressions. Allow the child to experiment with the concept themselves (F).
**Problem solving games**	Perseverance in the task and search for strategies			Above-mentioned objects and Shape sorter boxes, stacking rings, containers.	Insert shapes, flip the jar to find the correct shape hole. Straighten the pole where the rings should be placed. Gently guide the child's gestures, verbalizing the actions and any corrections needed to successfully complete the play tasks (F).
**Focused attention and recognition**	Recognizes specific figures in a book.		In the adult's arms.<br><br>On the mat.<br><br>At the table.	Books with pictures.	In a shared context of pleasurable play, the adult reads a story and shows the pictures to the child, naming them and helping the child recognize them (F).
			**18-24 months**		
**Ability**	**Objective**	**Context**	**Child**	**Tools**	**Proposals**
**Memory**	Removes 3 stickers from the face in front of the mirror.<br><br>Finds a hidden object under the correct cloth, even when the position	Quiet environment free of distracting or confusing factors (F).<br><br>Ecological context not specifically adapted (S).	Seated in the postural system or high chair.<br><br>Table with a recessed opening (F).<br><br>On the mat (S).	Stickers.<br><br>Building blocks, cubes, toys with wheels, objects that imitate everyday actions (e.g., pots, houses, doctor’s tools,	In a shared atmosphere of play and fun, propose engaging activities using the mirror.<br><br>Introduce simple play patterns of hiding, construction, alignment, and puzzle reconstruction by providing the adult's imitative model, then leaving the action incomplete to motivate the child

	is inverted (with visible movement).		Seated with a regular table (S).	personal care items), clothes, shape sorters, puzzles, shape sorters, brushes, and balls.<br><br>Doll.	to finish it (F).<br><br>Leave the play materials available for each request and wait for the child's initiative in organizing different play patterns (C).<br><br>In a shared atmosphere of pleasure and play, propose gestures, facial expressions, and actions within nursery rhymes and songs. After a few repetitions, stop and wait for the child's imitative initiative.<br><br>In a shared atmosphere of pleasure and play, propose practical, constructive, and functional games with increasing levels of difficulty, carefully balancing multiple variables: context, materials, actions, and roles (C). Always pay attention to the child's reactions, signs of frustration tolerance, and ensure the level of challenge is optimal for them.
**Practical-constructive play**	Stacks a tower of 3 cubes by 18 months, 5 cubes by 21 months, and 6 cubes by 24 months.<br><br>Aligns cubes to imitate a train.				
**Body schema**	Points to 5 body parts on a doll-puzzle.				
**Imitation**	Simple imitations of the adult.				
**Cause-and-effect exploration play**	Pulls a string to make a toy with wheels move.				
**Game of exploring spatial relationships**	Place the circle and square in their respective holes.<br><br>By 24 months, place 3 simple shapes in the molds.	Quiet environment free of distracting or confusing factors (F).<br><br>Ecological context not specifically adapted (S).	Seated in the postural system or high chair.<br><br>Table with a recessed opening (F).<br><br>On the mat (S).<br><br>Seated with a regular table (S).	Shape sorters, building blocks, cubes, toys with wheels, objects that mimic everyday actions (e.g., pots, houses, doctor tools, personal care items), interlocking shapes, and shape sorters.	Propose and leave various materials available for the child, waiting for their initiatives to interact with the play. Observe their attempts, possible mistakes, and organizational difficulties.<br><br>Gently guide the child's gestures, verbalize the actions, and offer any necessary corrections to successfully complete the play activities (F).<br><br>Offer play activities with increasing difficulty levels (C).
**Problem solving games**	Perseverance in the task and search for strategies				
